# Supplementary material for: Ugi Reaction on α-Phosphorated Ketimines for the Synthesis of Tetrasubstituted α-Aminophosphonates and Their Applications as Antiproliferative Agents
Source: Molecules. 2021 Mar 16;26(6):1654. doi: 10.3390/molecules26061654 (PMC8002371; doi:10.3390/molecules26061654)

molecules

Supporting Information

Ugi reaction on α-phosphorated ketimines for the synthesis of tetrasubstituted α-aminophosphonates and their applications as antiproliferative agents.

Adrián López-Francés,[1] Xabier del Corte,[1] Edorta Martinez de Marigorta,[1] Francisco Palacios[1,*] and Javier Vicario[1,*]

[1] Departamento de Química Orgánica I, Centro de Investigación y Estudios Avanzados "Lucio Lascaray"- Facultad de Farmacia, University of the Basque Country, UPV/EHU Paseo de la Universidad 7, 01006 Vitoria-Gasteiz, SPAIN

* Correspondence: francisco.palacios@ehu.eus (F. P.) / javier.vicario@ehu.eus (J. V.)

Dimethyl (2-(cyclohexylamino)-1-((4-methylphenyl)sulfonamido)-2-oxo-1-phenylethyl)phosphonate (13a).

¹H NMR (400 MHz, $CDCl_3$)

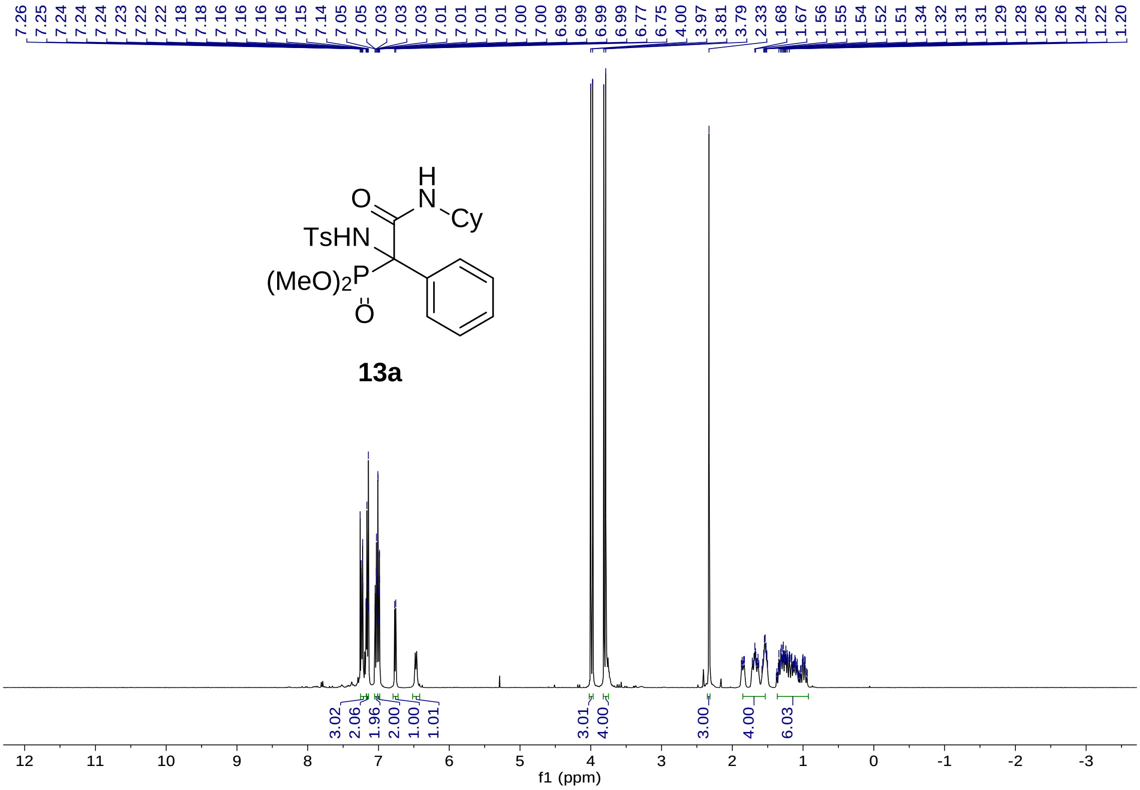

¹³C NMR {1H} (101 MHz, $CDCl_3$)

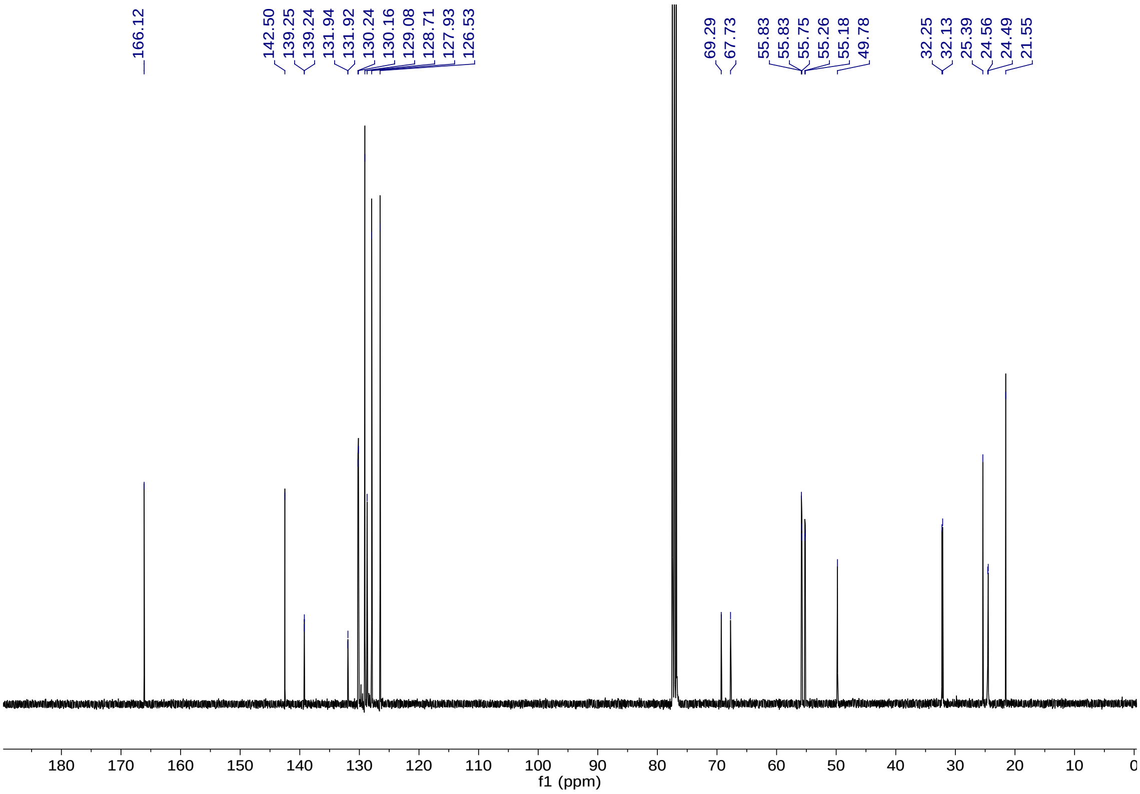

^{31}P NMR (121 MHz, CDCl3)

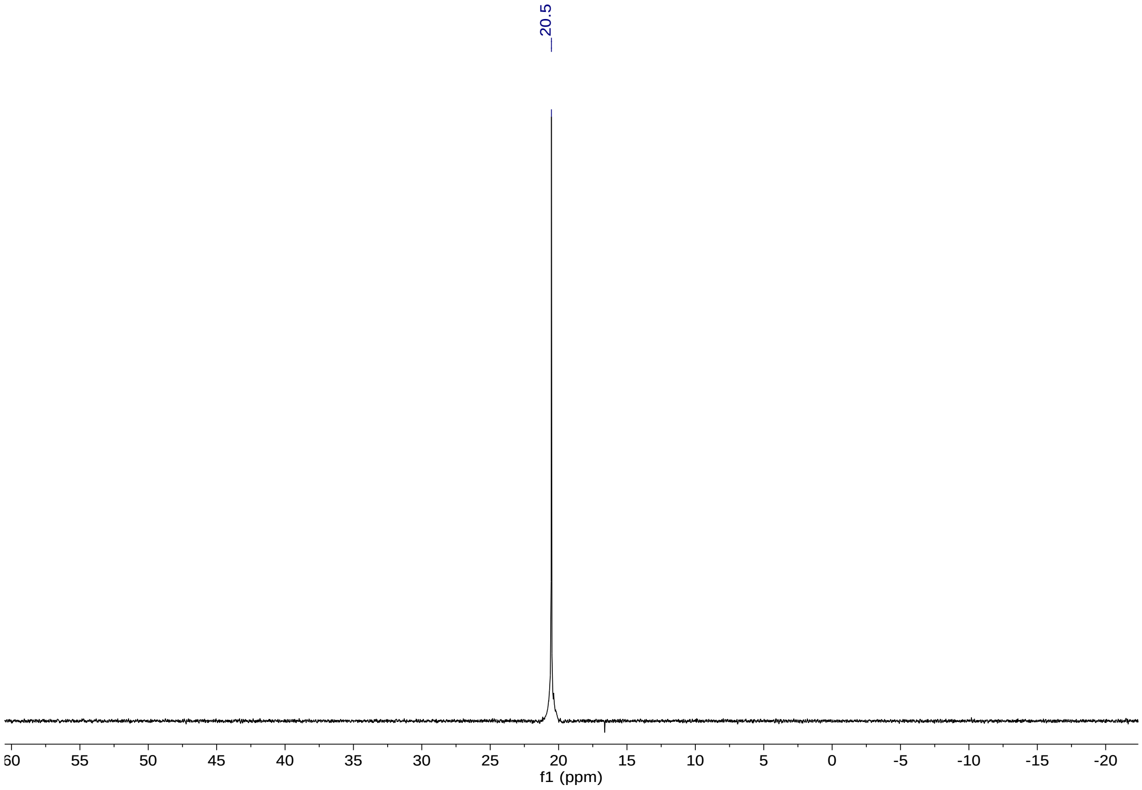

Methyl (2-(dimethoxyphosphoryl)-2-((4-methylphenyl)sulfonamido)-2-phenylacetyl)glycinate (13b)

^{1}H NMR (400 MHz, $CDCl_3$)

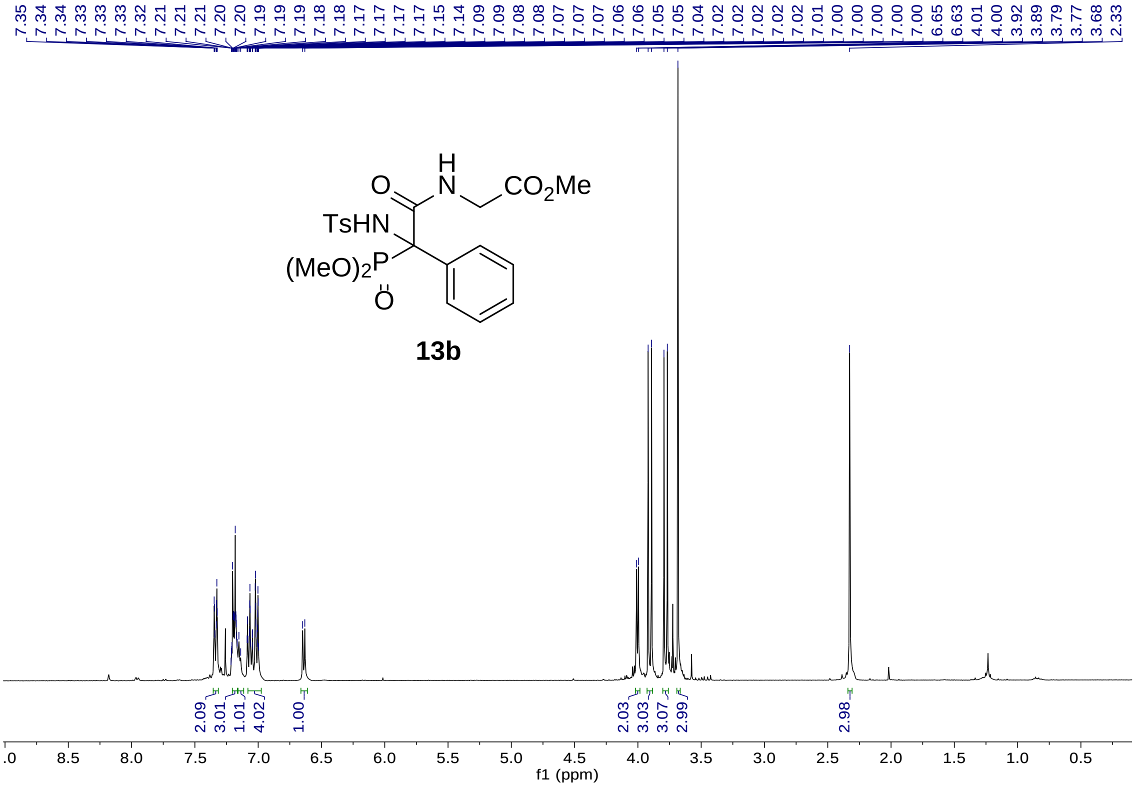

^{13}C NMR {^{1}H} (101 MHz, $CDCl_3$)

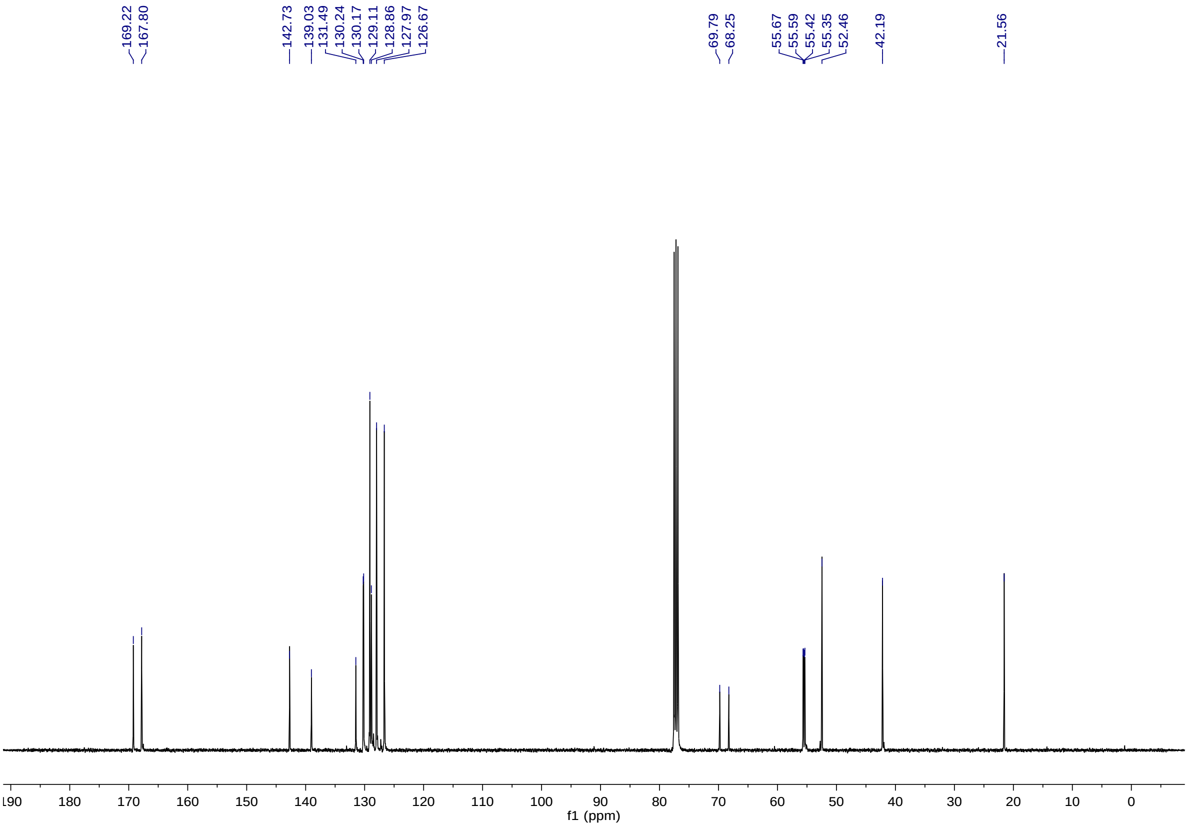

^{31}P NMR (121 MHz, CDCl3)

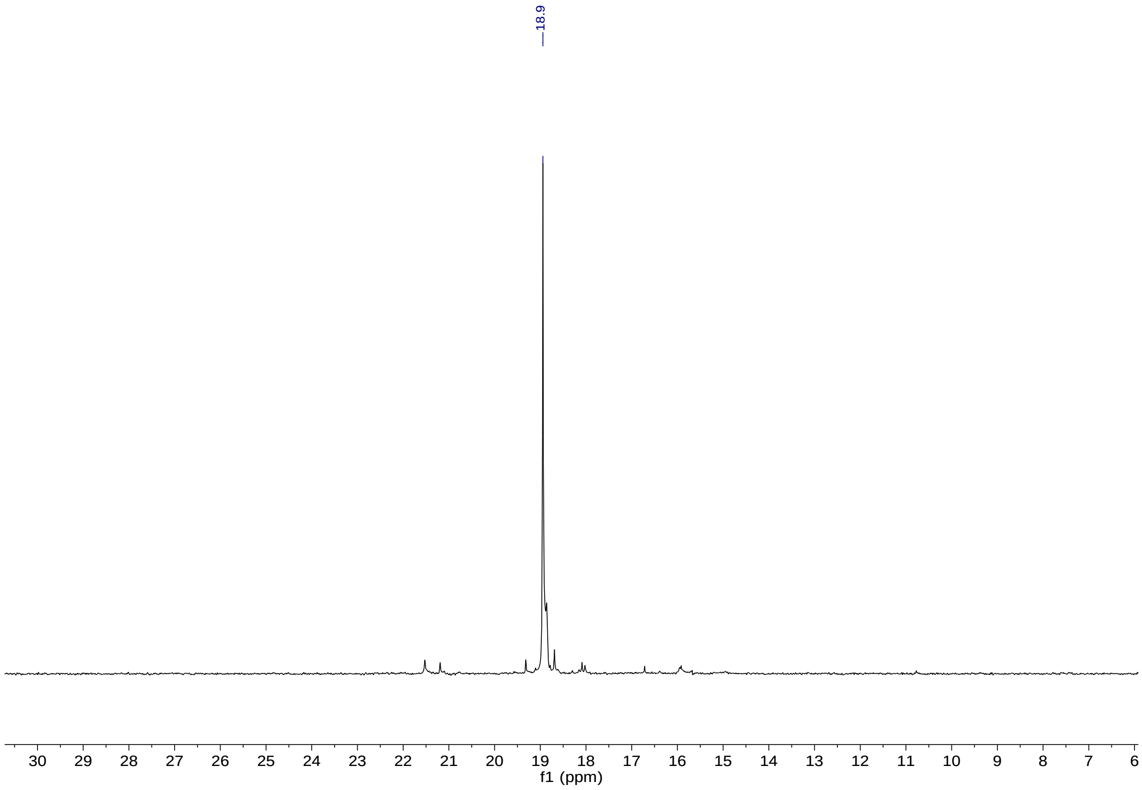

Dimethyl (2-(benzylamino)-1-((4-methylphenyl)sulfonamido)-2-oxo-1-phenylethyl)phosphonate (13c)

^{1}H NMR (400 MHz, CDCl3)

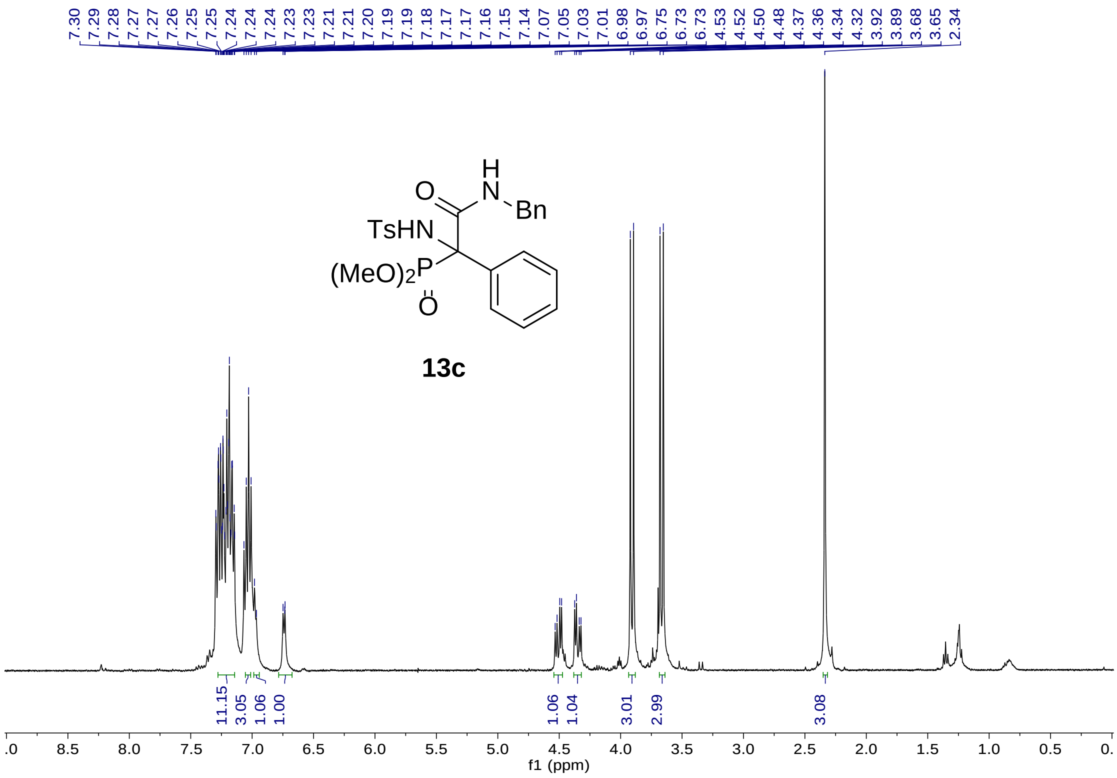

^{13}C NMR {^{1}H} (101 MHz, CDCl$_3$)

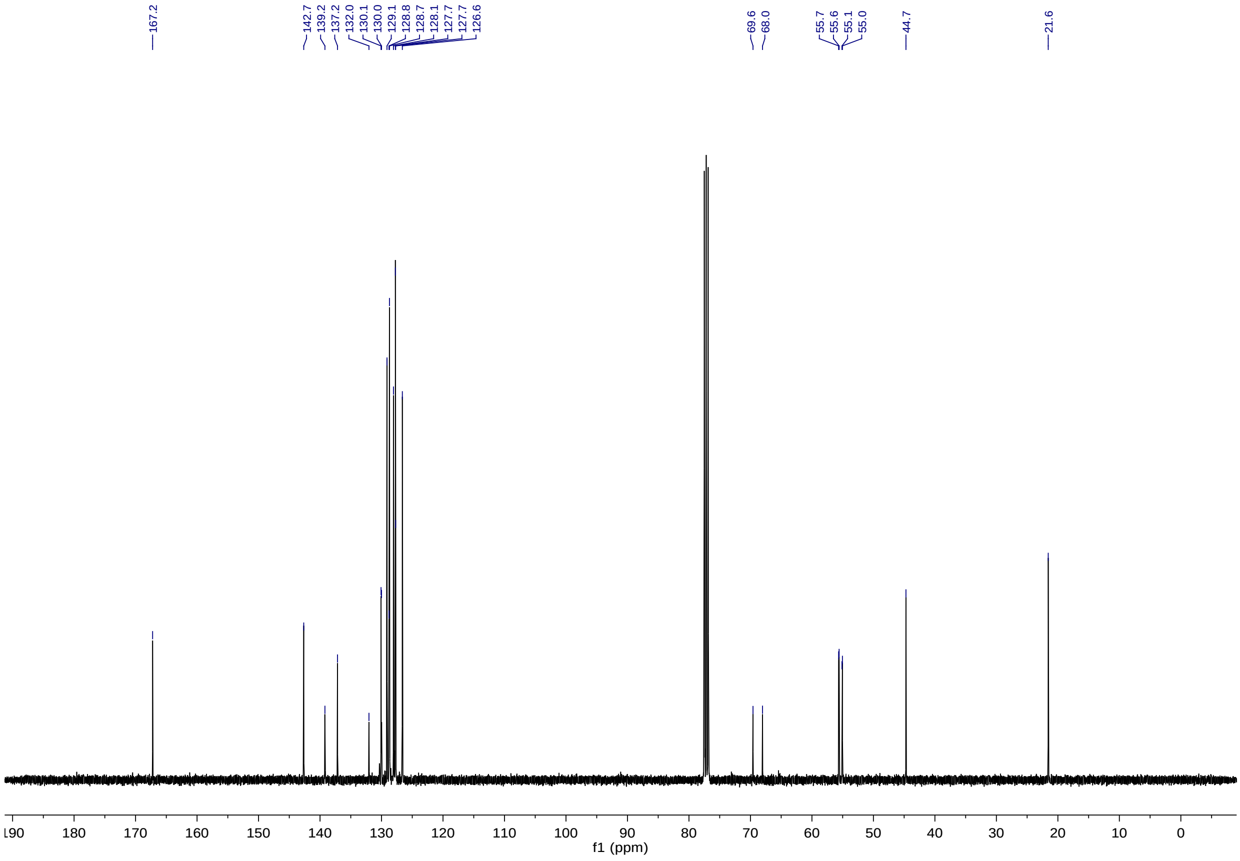

^{31}P NMR (121 MHz, CDCl3)

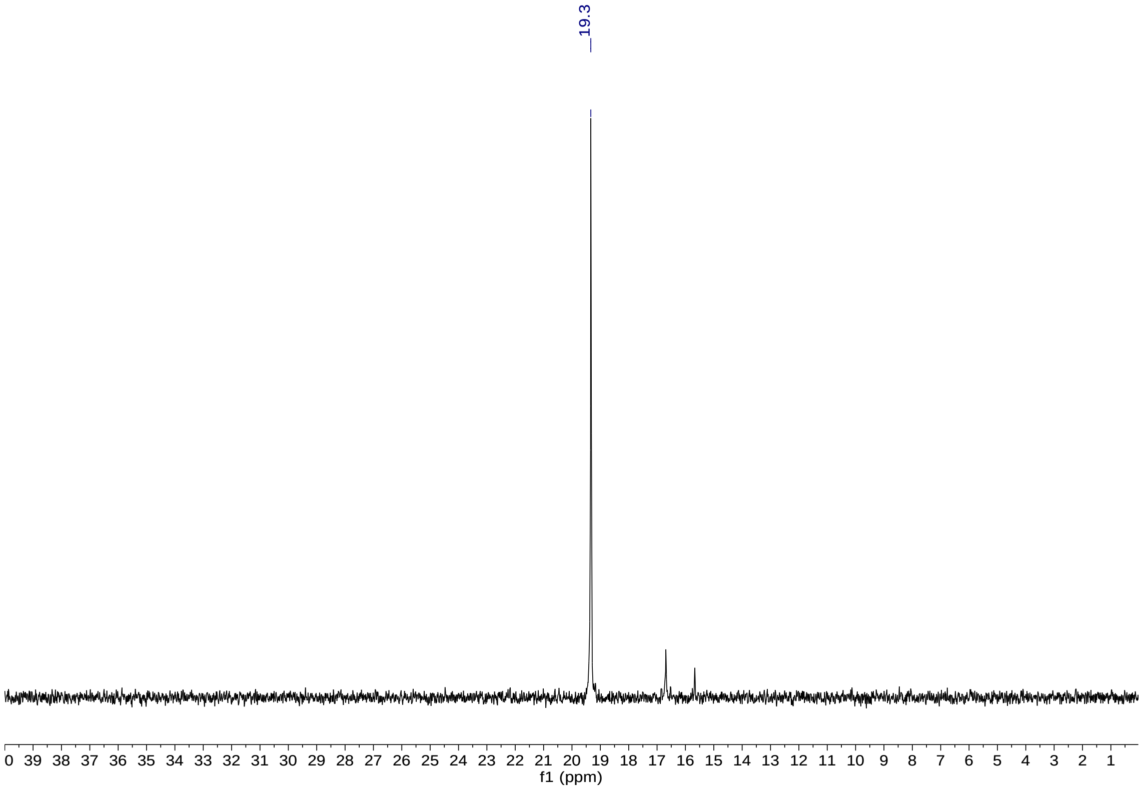

Diethyl (2-(cyclohexylamino)-1-((4-methylphenyl)sulfonamido)-2-oxo-1-phenylethyl)phosphonate (13d)

^{1}H NMR (400 MHz, CDCl3)

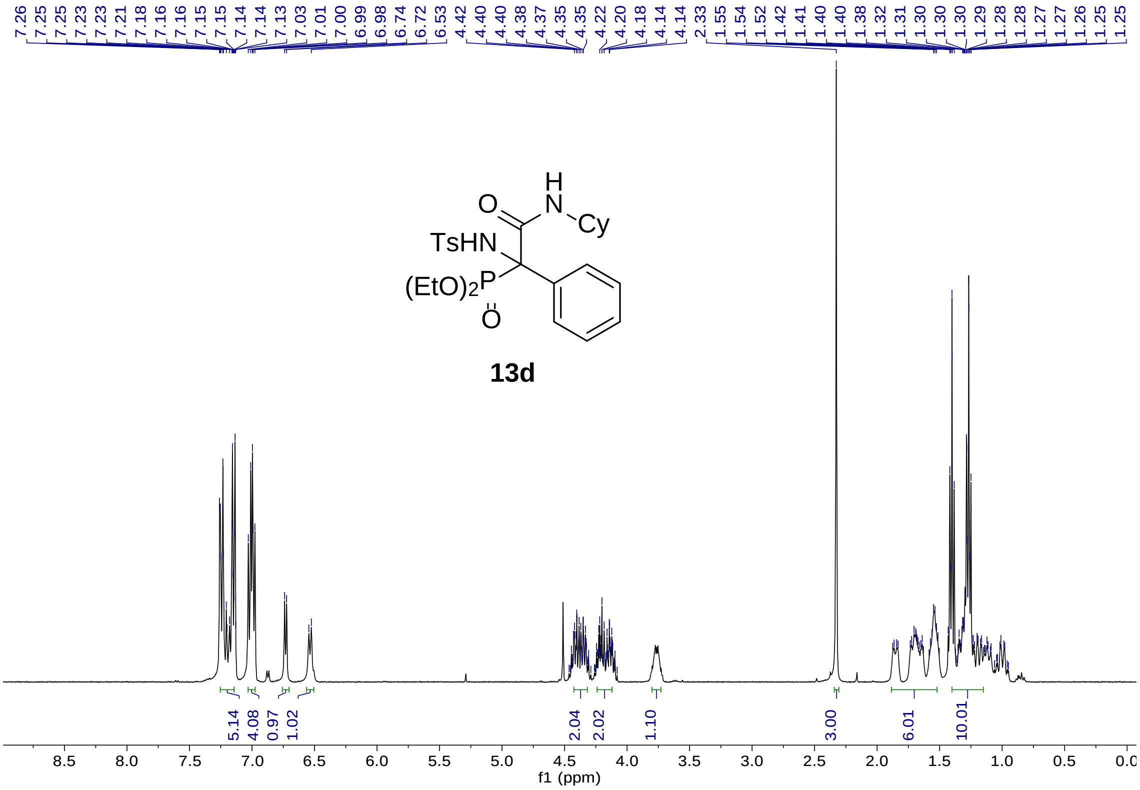

^{13}C NMR {^{1}H} (101 MHz, CDCl3)

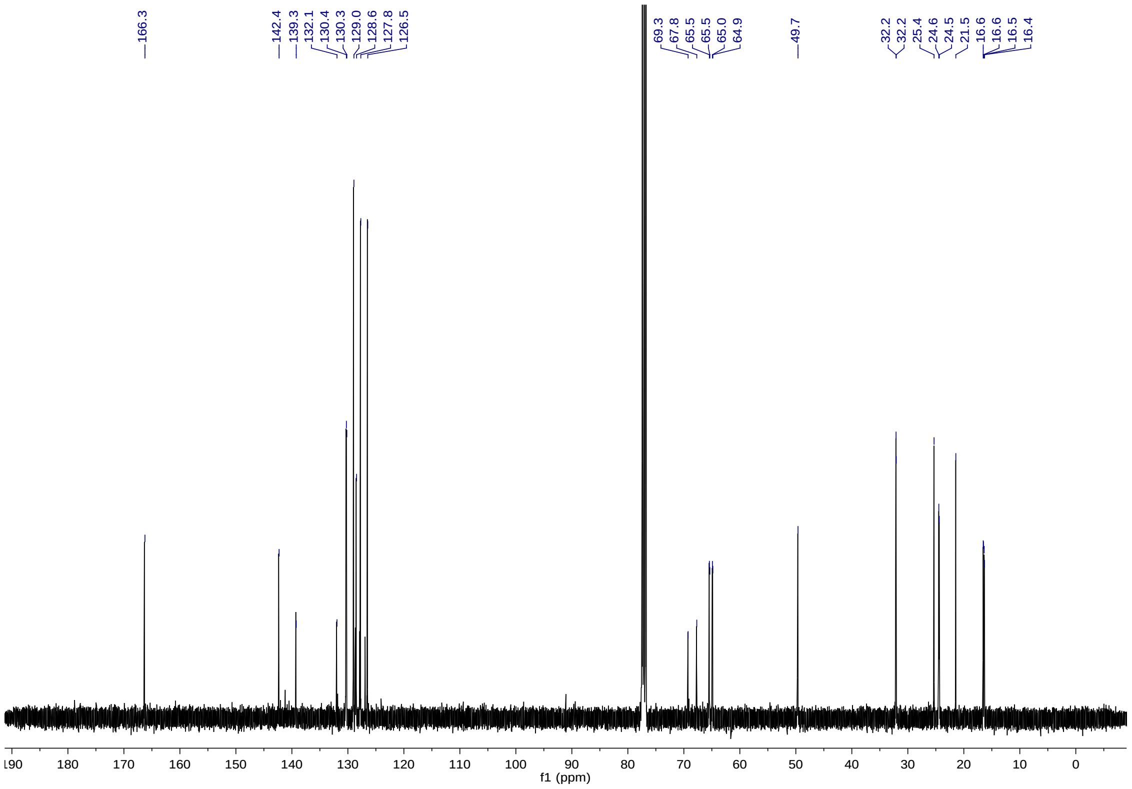

^{31}P NMR (121 MHz, CDCl3)

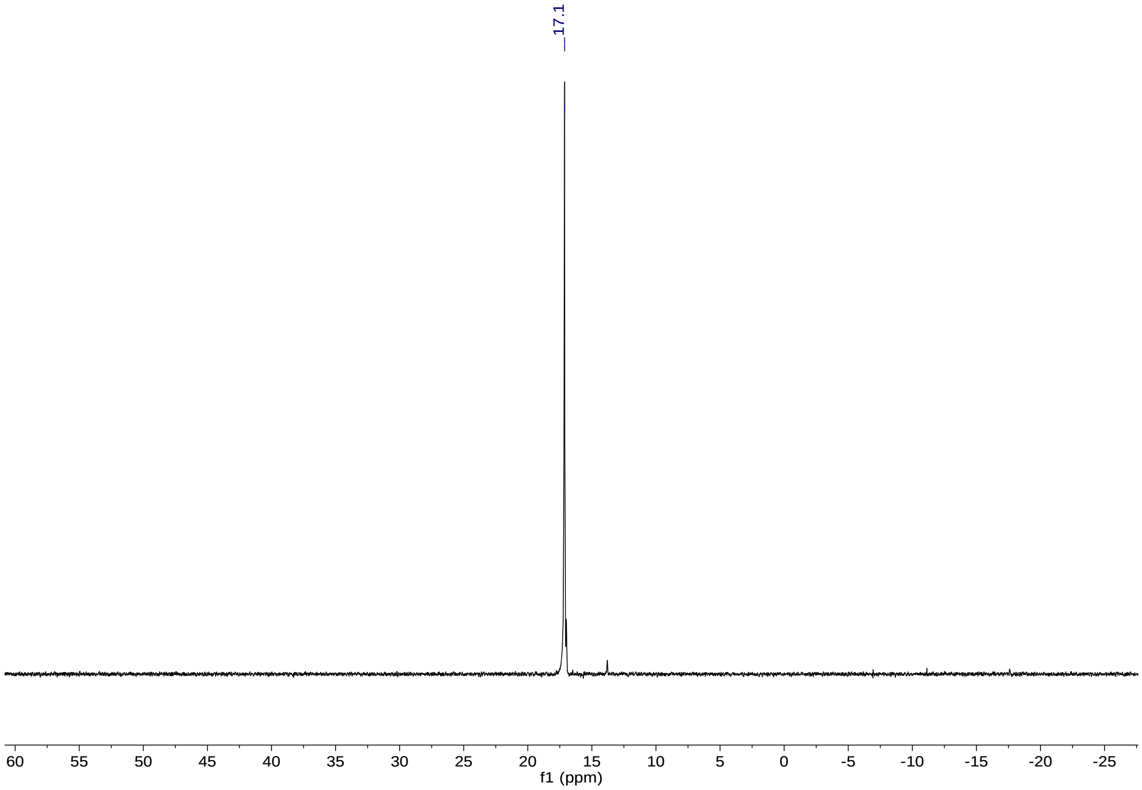

Methyl (2-(diethoxyphosphoryl)-2-((4-methylphenyl)sulfonamido)-2-phenylacetyl)glycinate (13e)

¹H NMR (400 MHz, CDCl3)

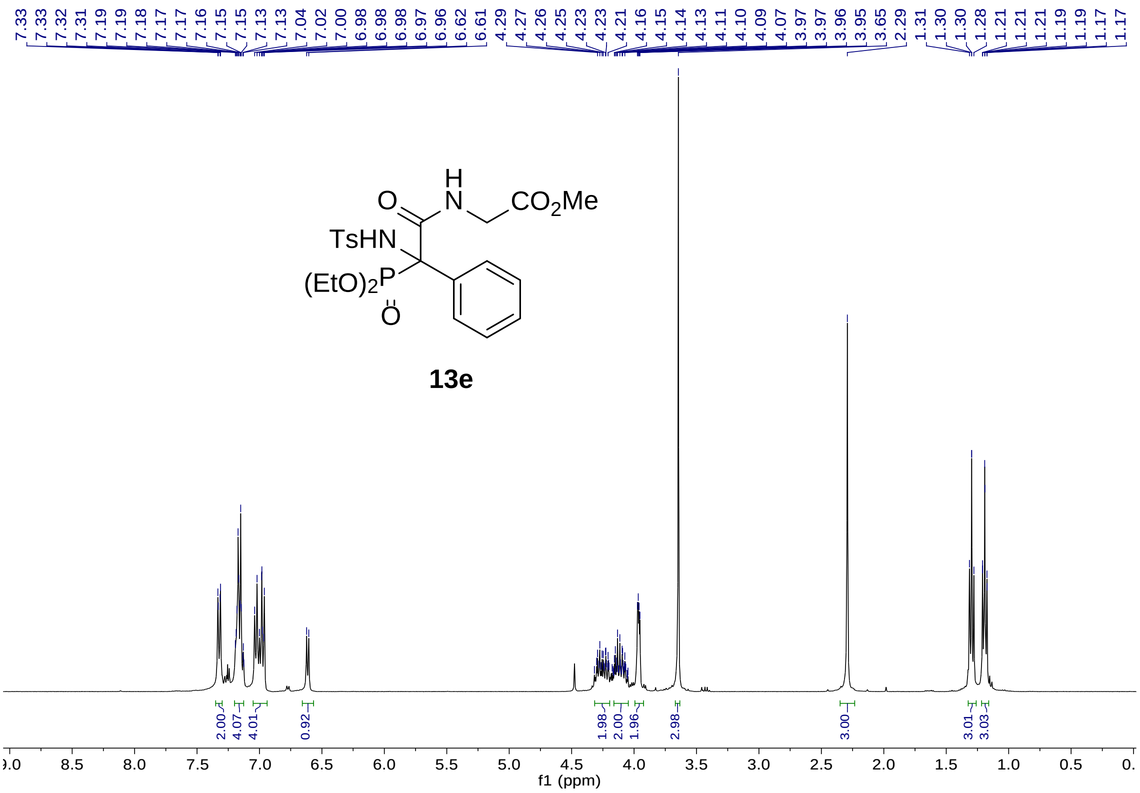

¹³C NMR {¹H} (101 MHz, CDCl₃)

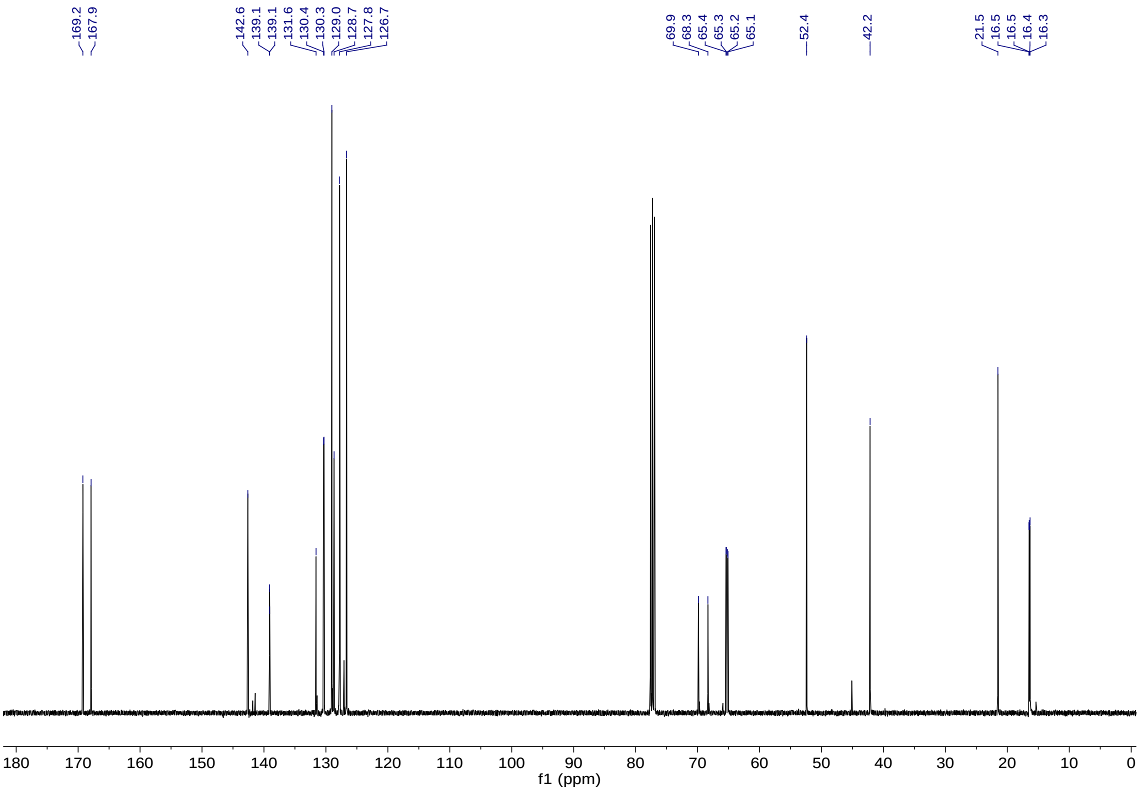

^{31}P-NMR (121 MHz, CDCl3)

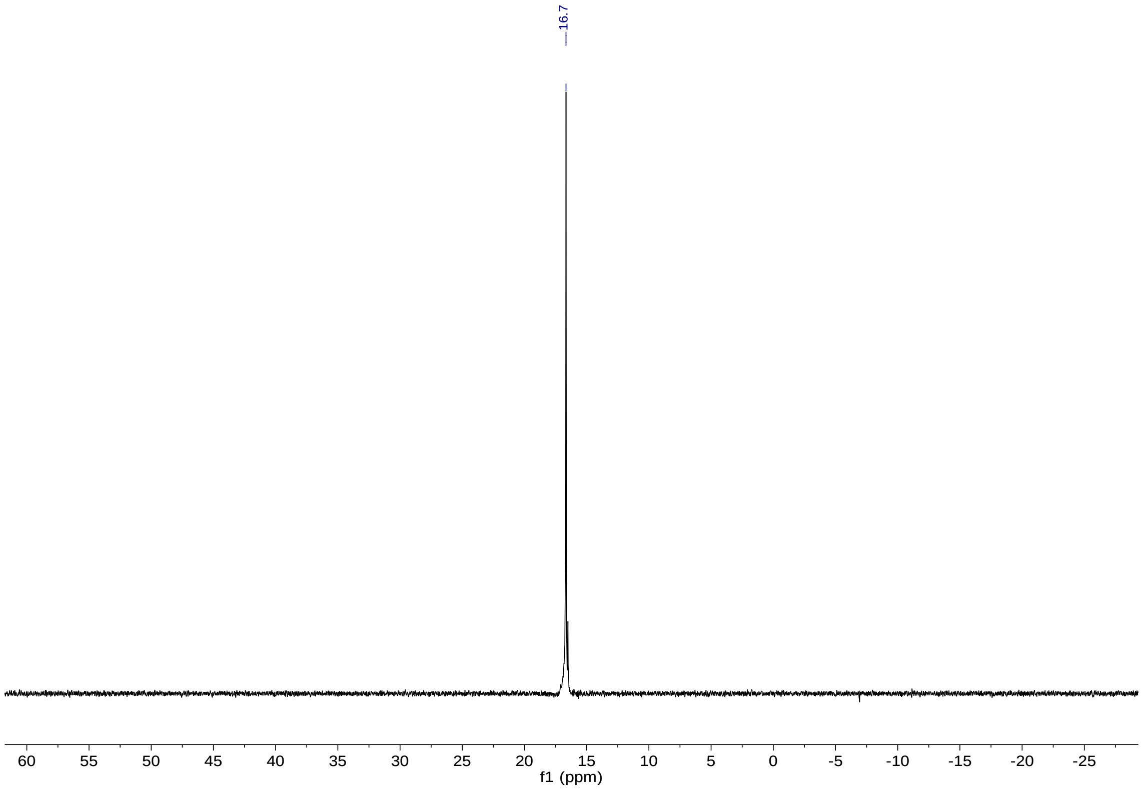

Diethyl (2-(benzylamino)-1-((4-methylphenyl)sulfonamido)-2-oxo-1-phenylethyl)phosphonate (13f)

¹H NMR (400 MHz, CDCl3)

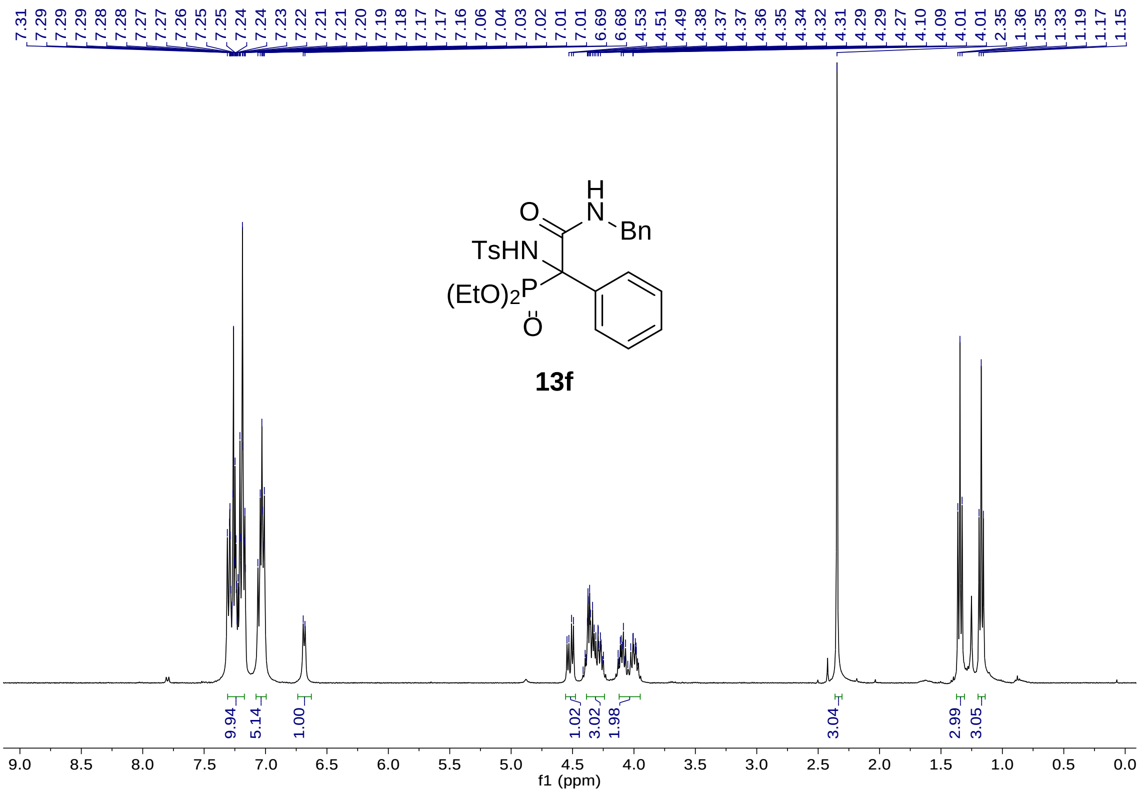

¹³C NMR {¹H} (101 MHz, $CDCl_3$)

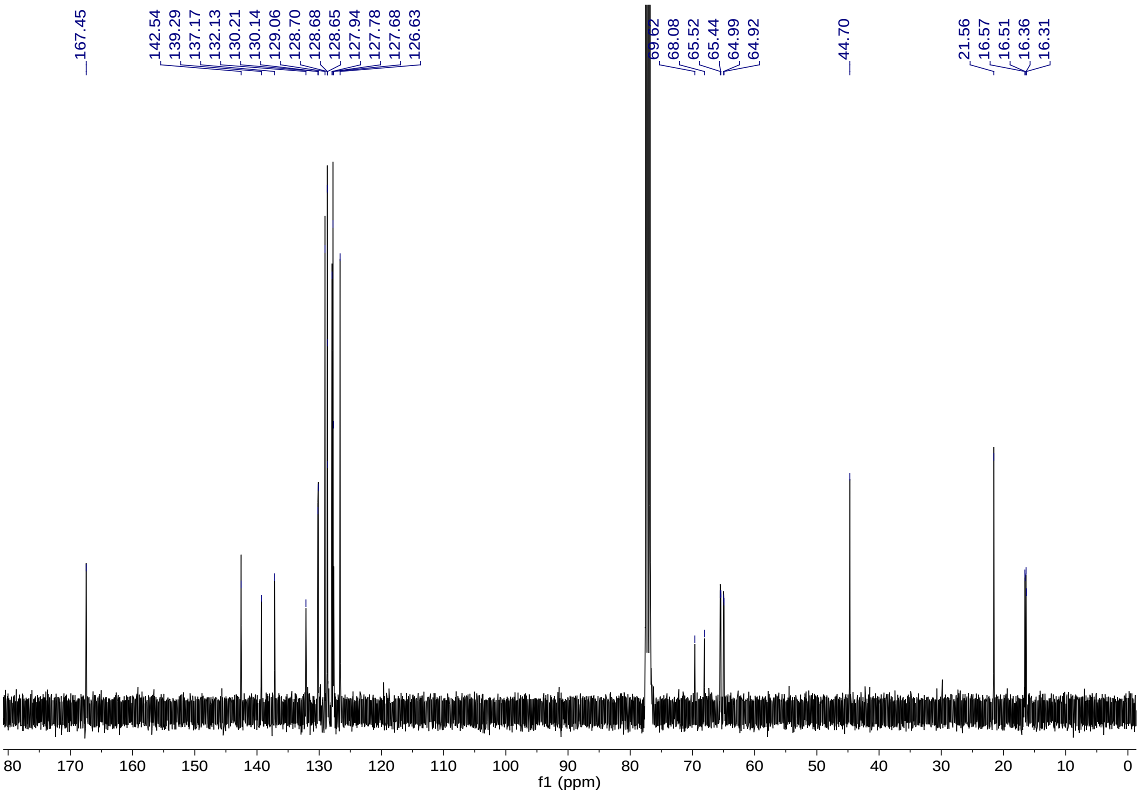

^{31}P-NMR (121 MHz, CDCl3)

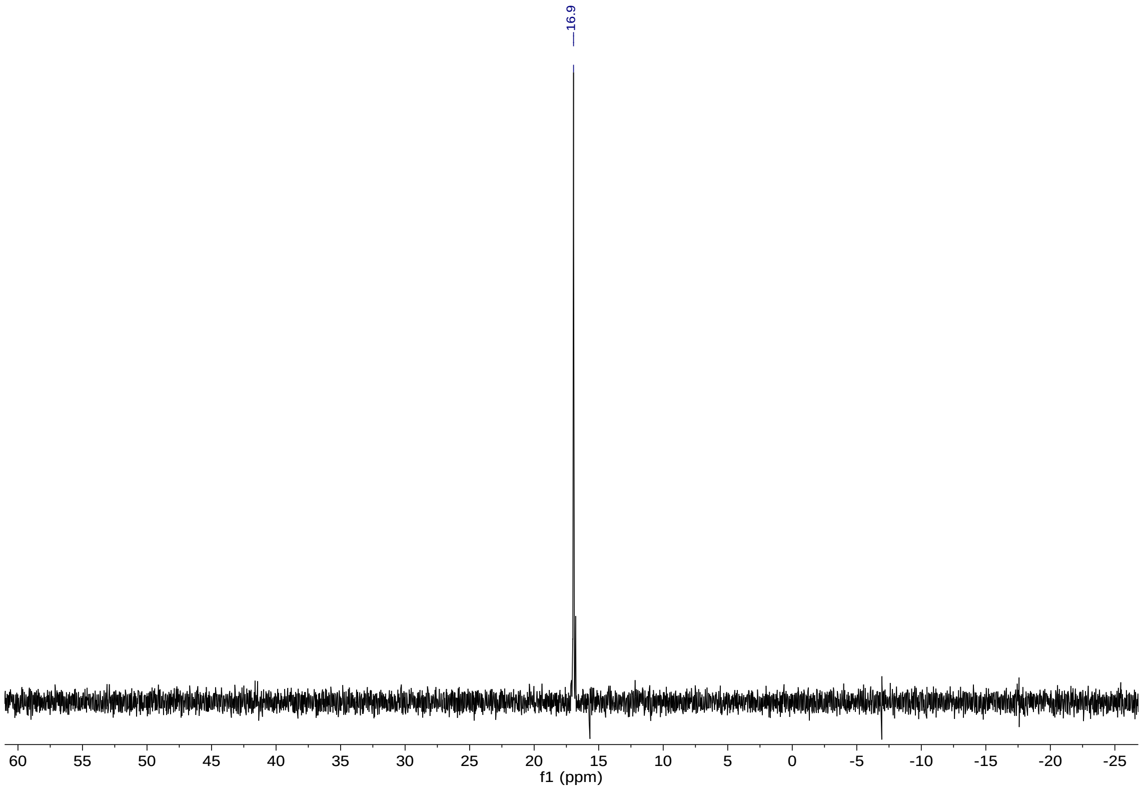

Dibenzyl (2-(cyclohexylamino)-1-((4-methylphenyl)sulfonamido)-2-oxo-1-phenylethyl)phosphonate (13g)

^{1}H NMR (400 MHz, $CDCl_3$)

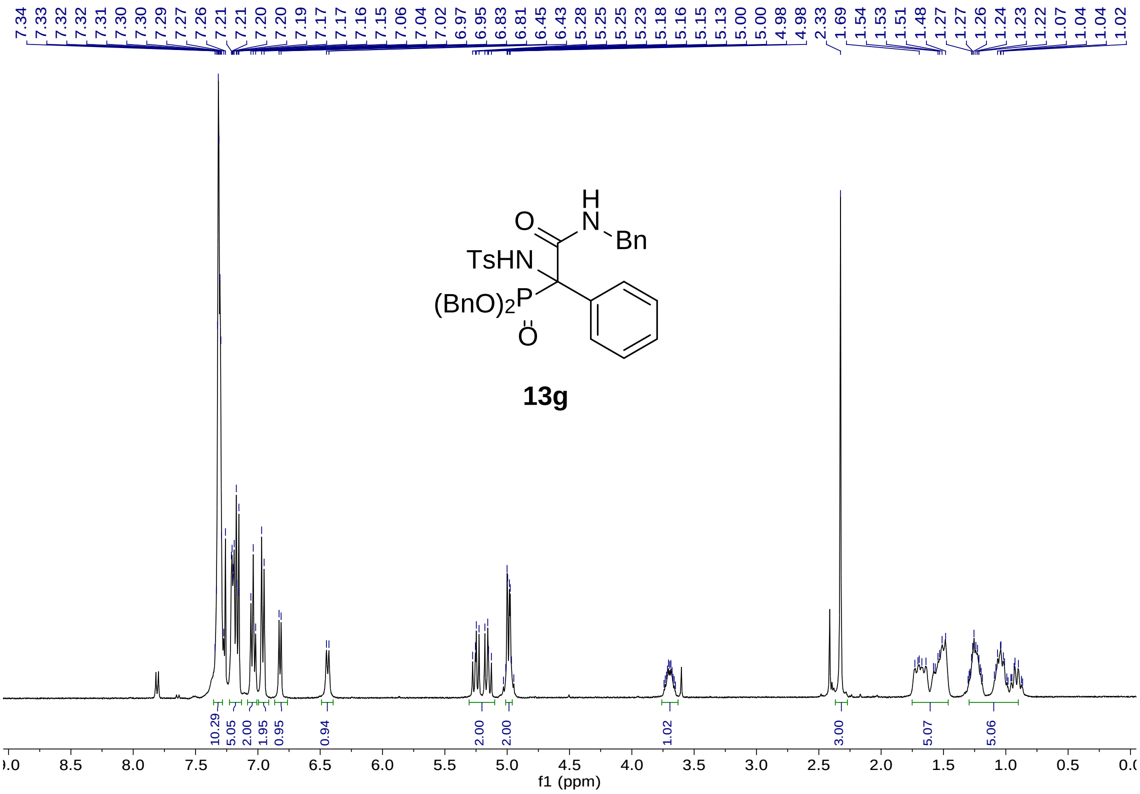

^{13}C NMR {1H} (101 MHz, $CDCl_3$)

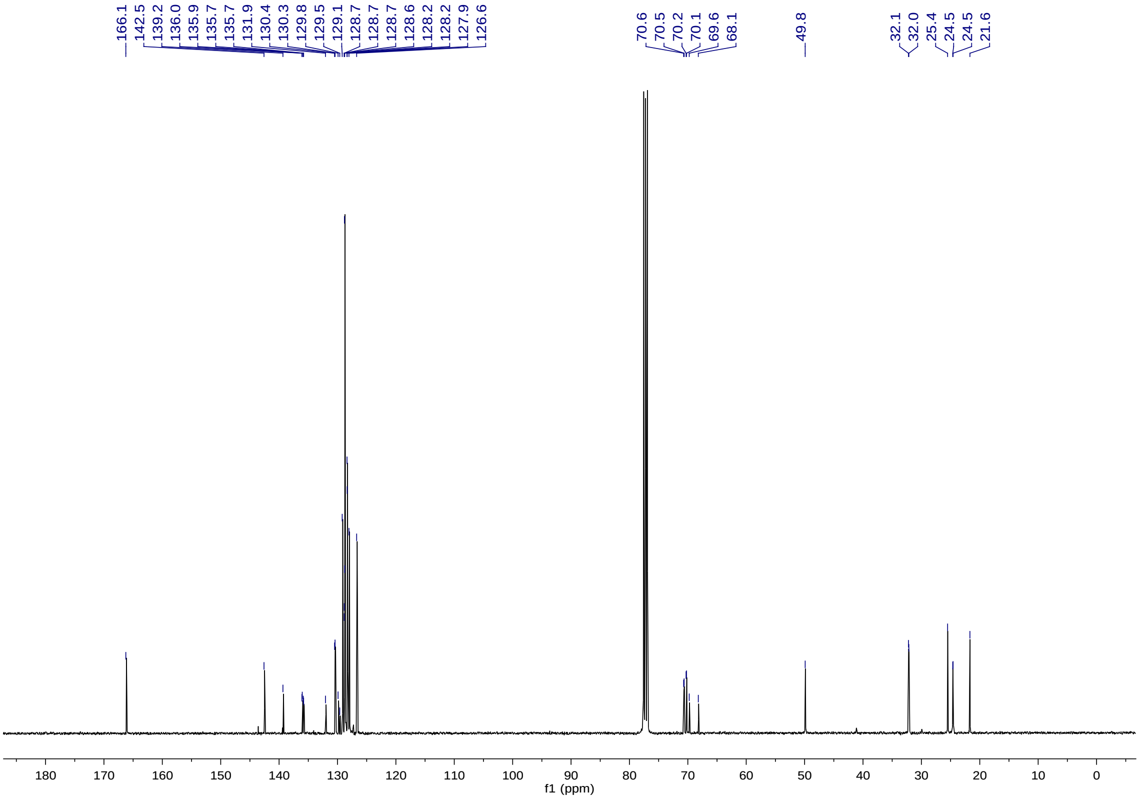

^{31}P-NMR (121 MHz, CDCl$_3$)

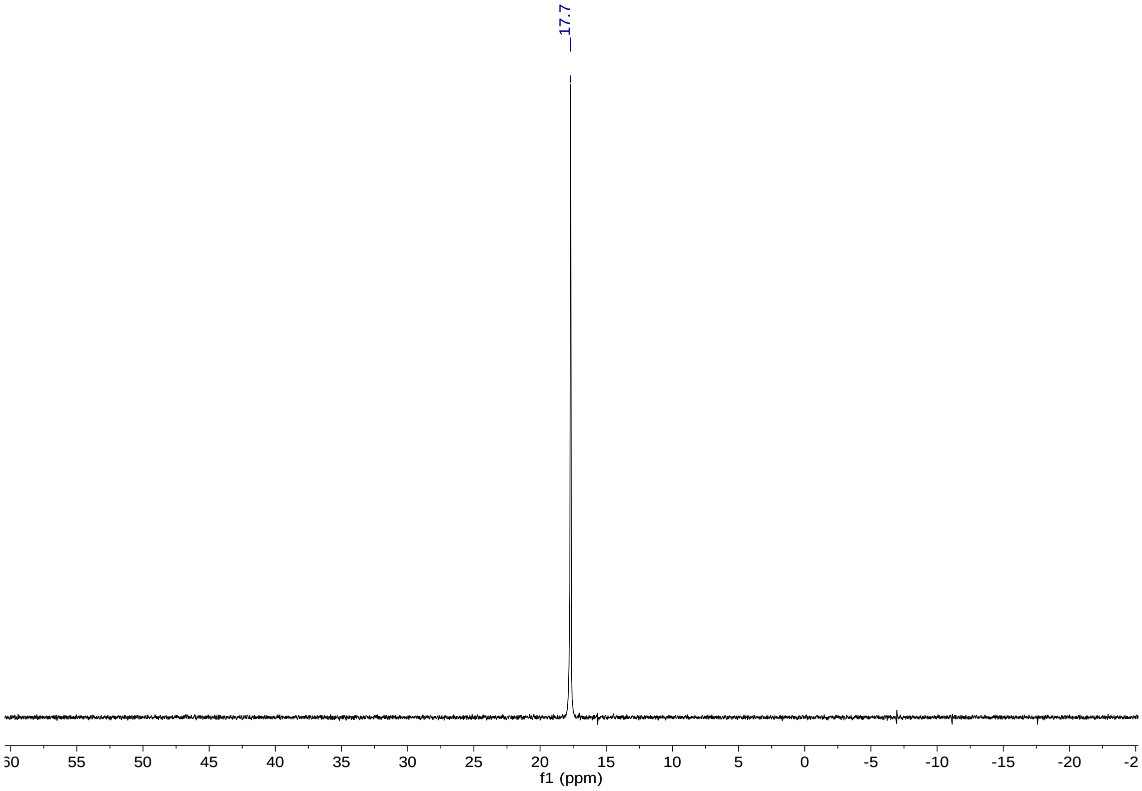

Diisopropyl (2-(cyclohexylamino)-1-((4-methylphenyl)sulfonamido)-2-oxo-1-phenylethyl)phosphonate (13h)

^{1}H NMR (400 MHz, $CDCl_3$)

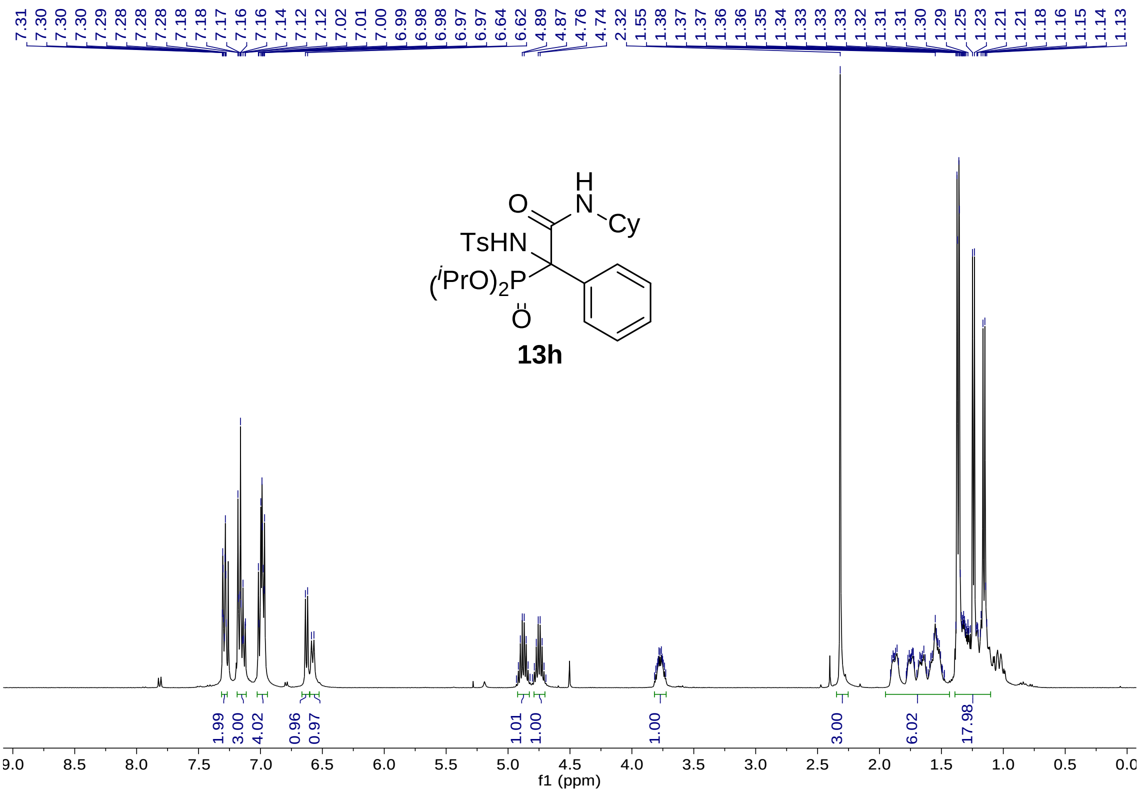

^{13}C NMR {^{1}H} (101 MHz, $CDCl_3$)

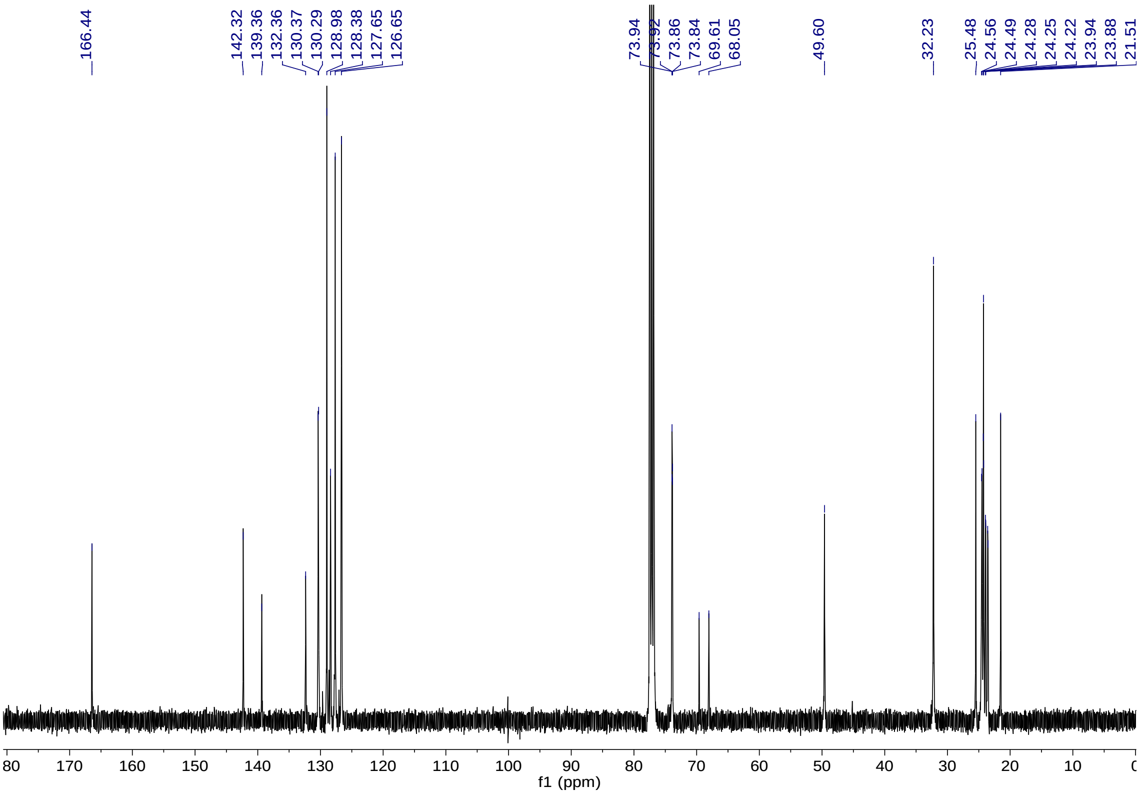

^{31}P NMR (121 MHz, $CDCl_3$)

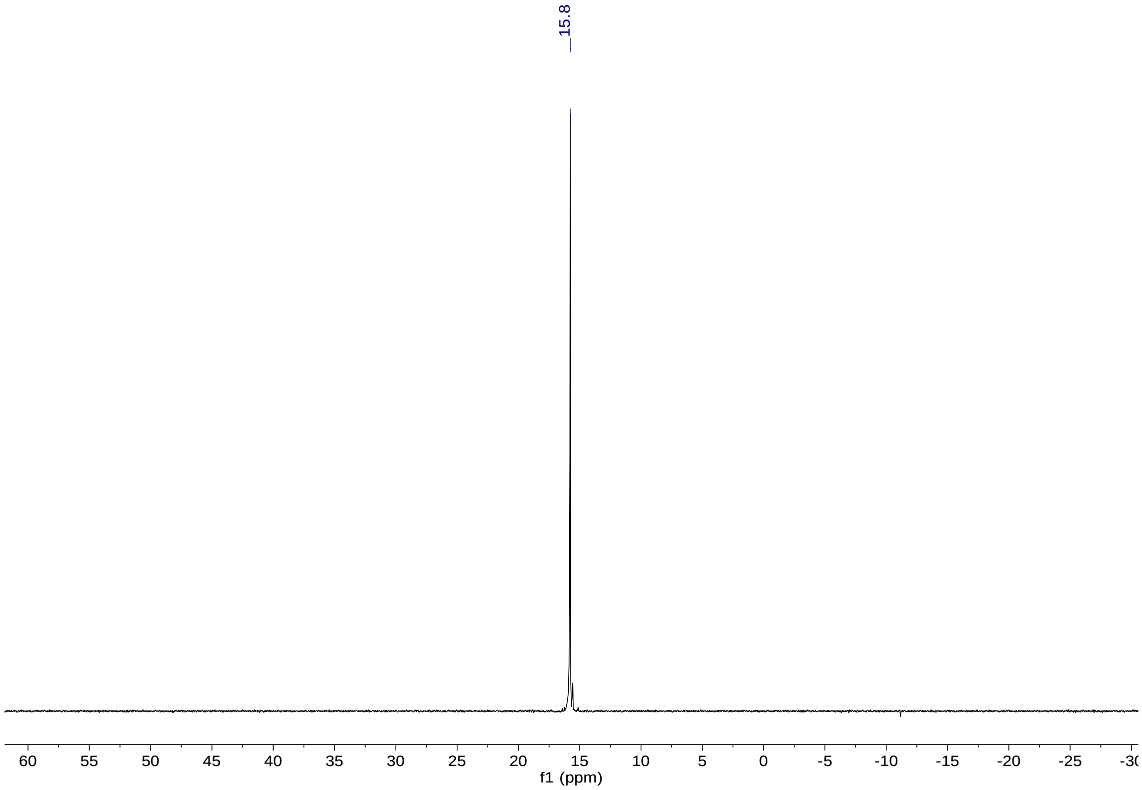

Methyl (2-(diisopropoxyphosphoryl)-2-((4-methylphenyl)sulfonamido)-2-phenylacetyl)glycinate (13i)

^{1}H NMR (400 MHz, $CDCl_3$)

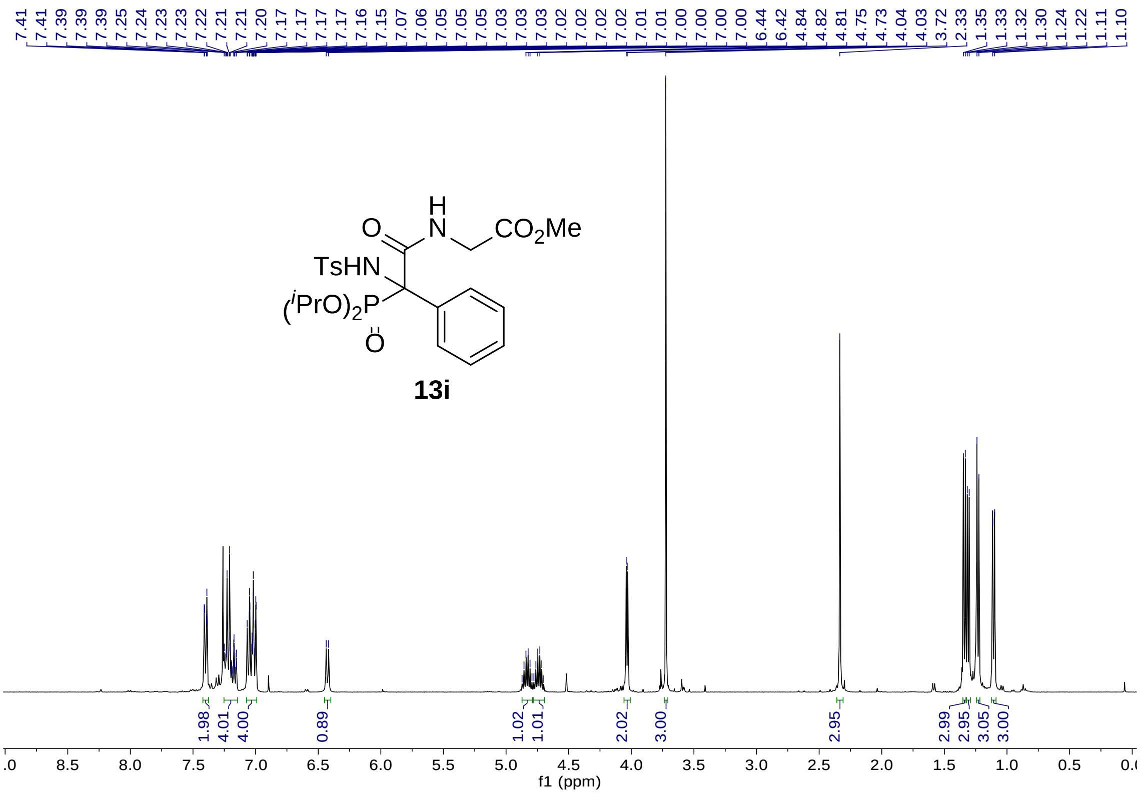

^{13}C NMR {^{1}H} (101 MHz, $CDCl_3$)

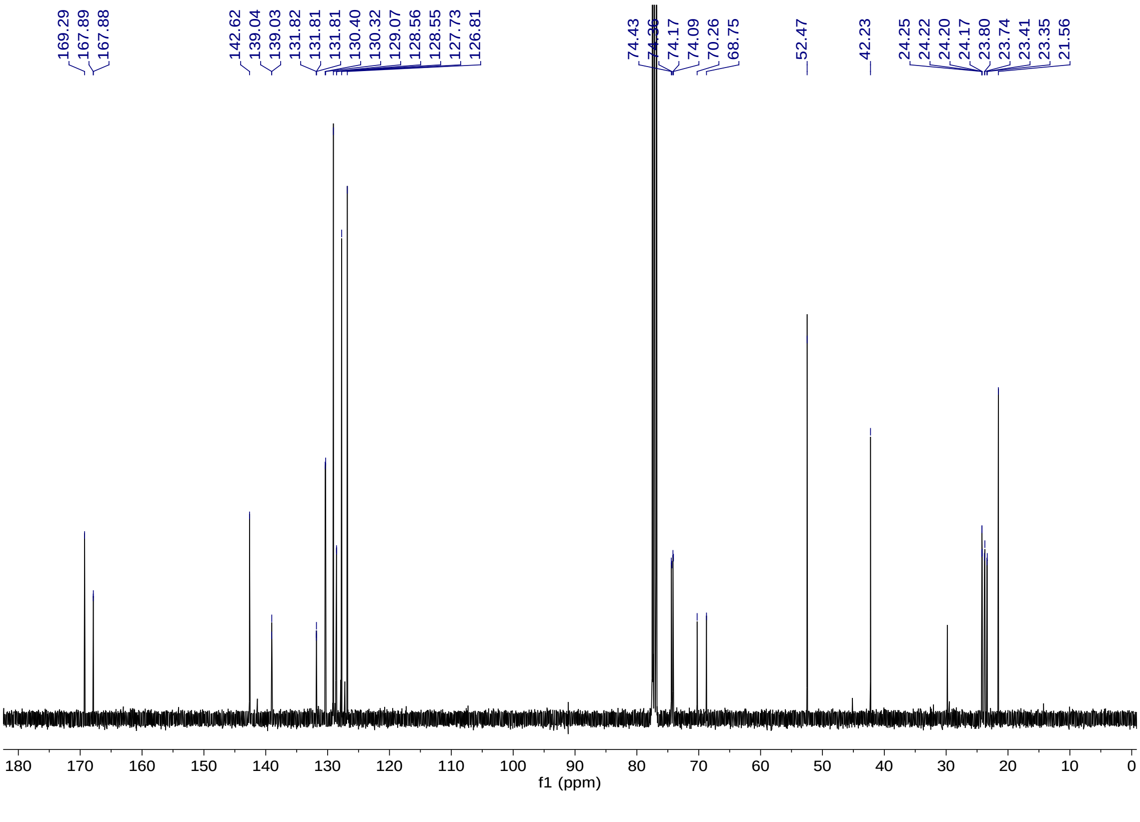

^{31}P NMR (121 MHz, $CDCl_3$)

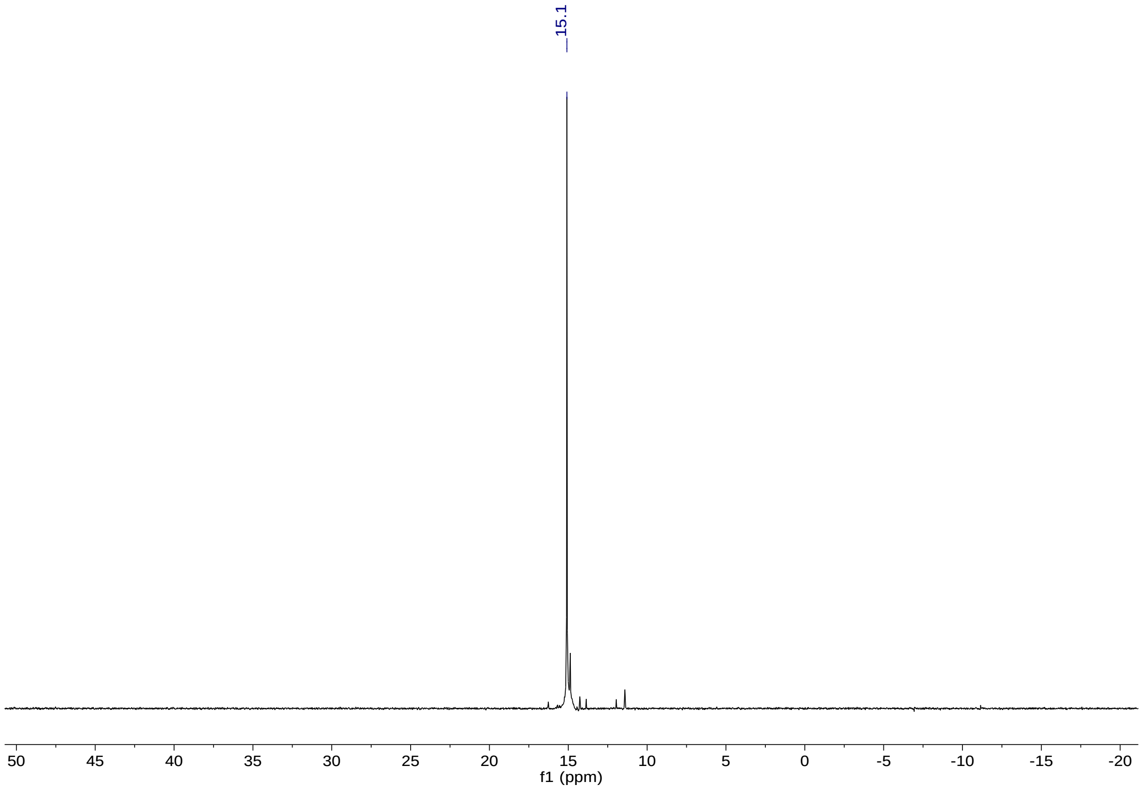

Diisopropyl (2-(benzylamino)-1-((4-methylphenyl)sulfonamido)-2-oxo-1-phenylethyl)phosphonate (13j)

^{1}H NMR (400 MHz, $CDCl_3$)

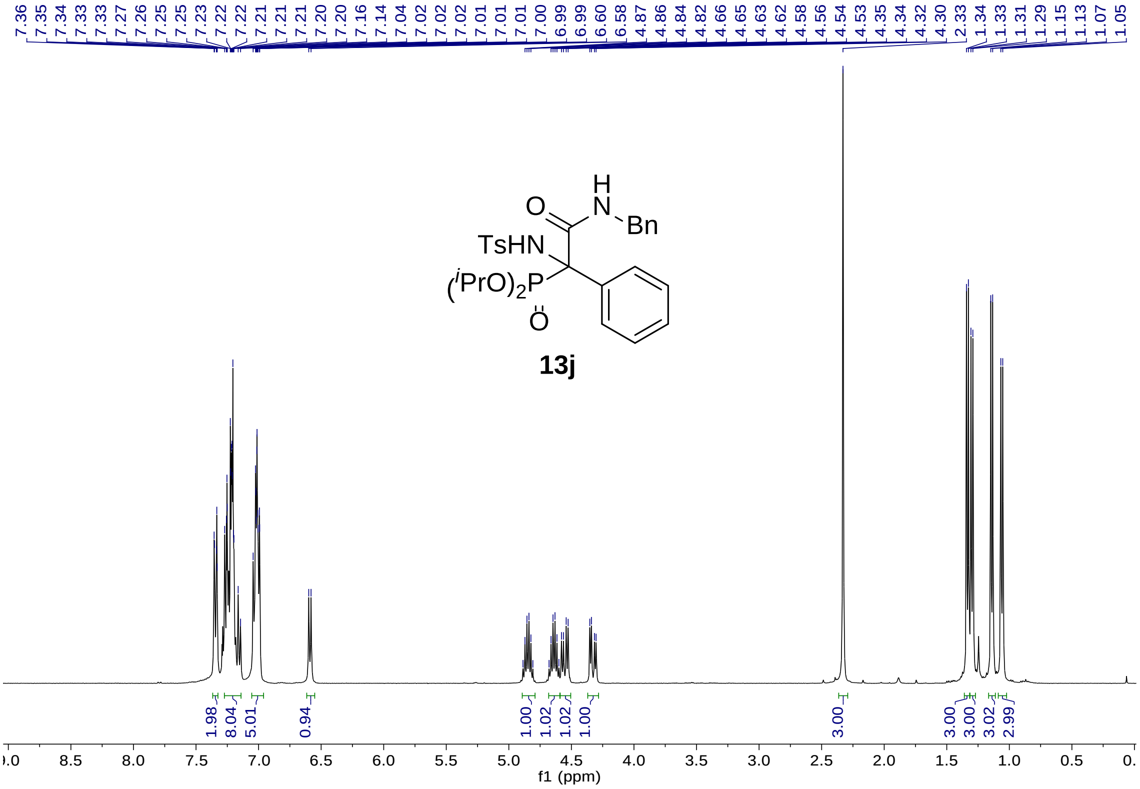

^{13}C NMR {^{1}H} (101 MHz, $CDCl_3$)

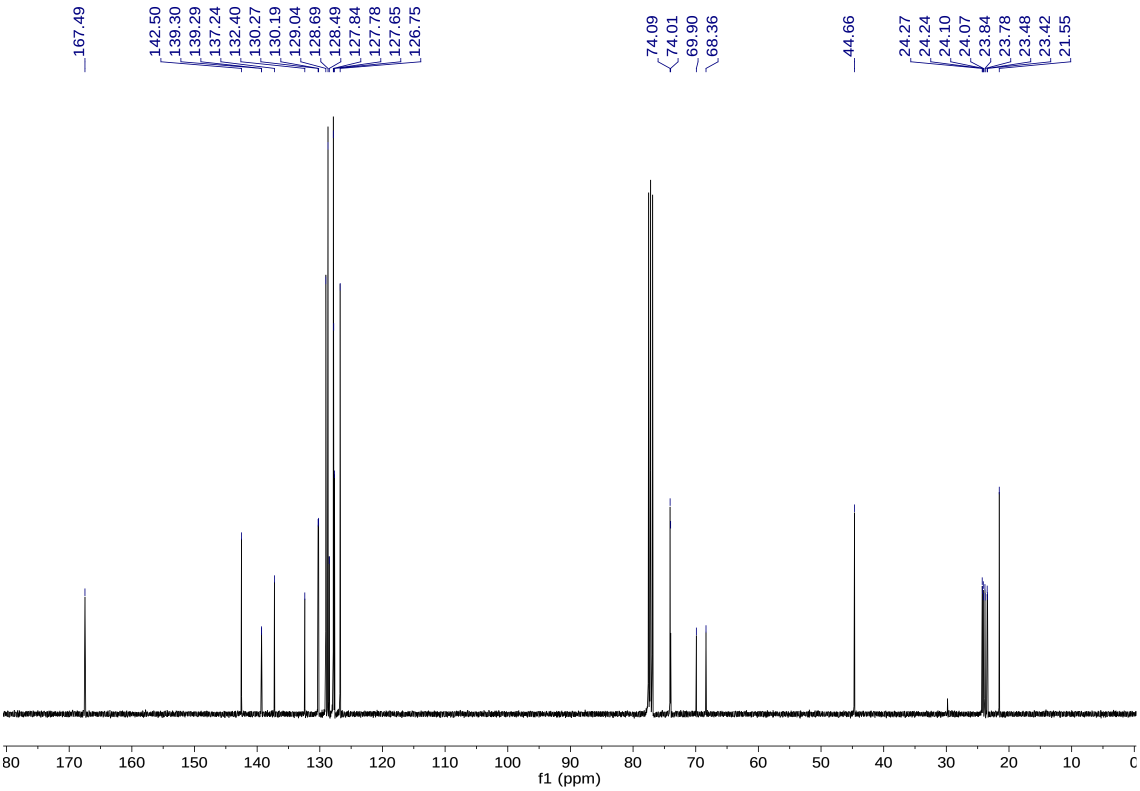

^{31}P NMR (121 MHz, $CDCl_3$)

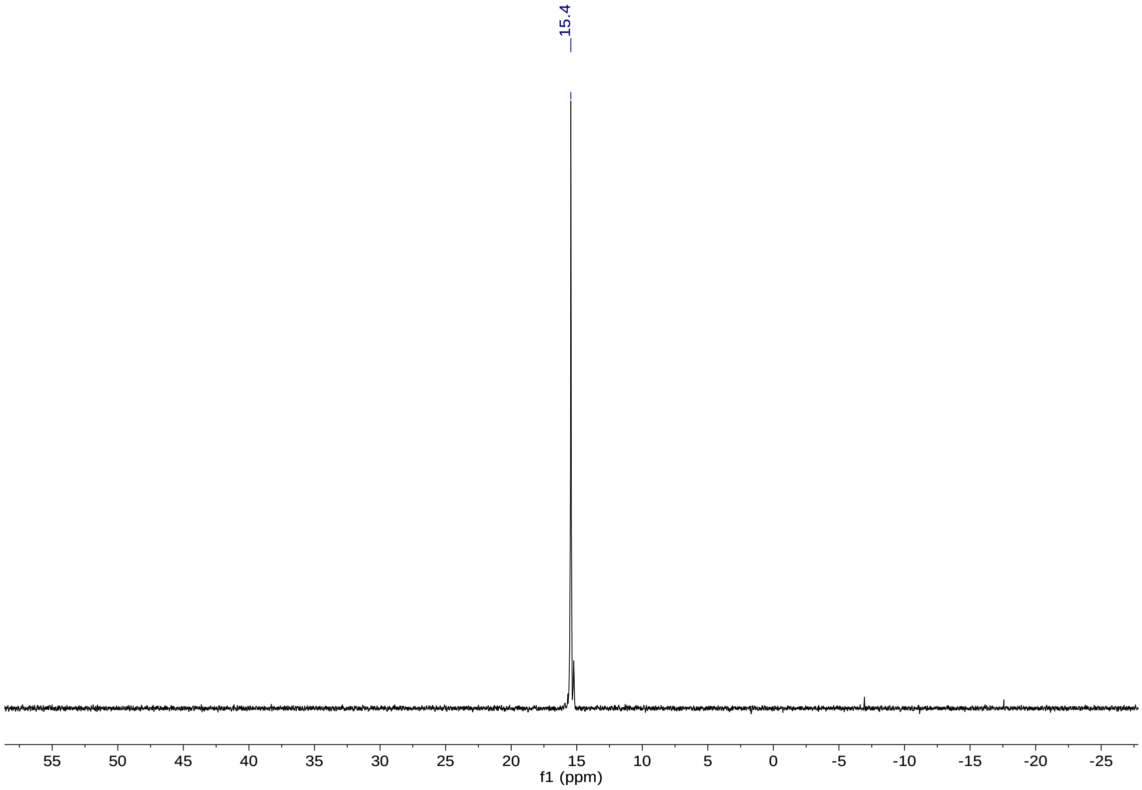

Dimethyl (2-(cyclohexylamino)-1-((4-methylphenyl)sulfonamido)-1-(4-nitrophenyl)-2-oxoethyl)phosphonate (13k)

^{1}H NMR (400 MHz, $CDCl_3$)

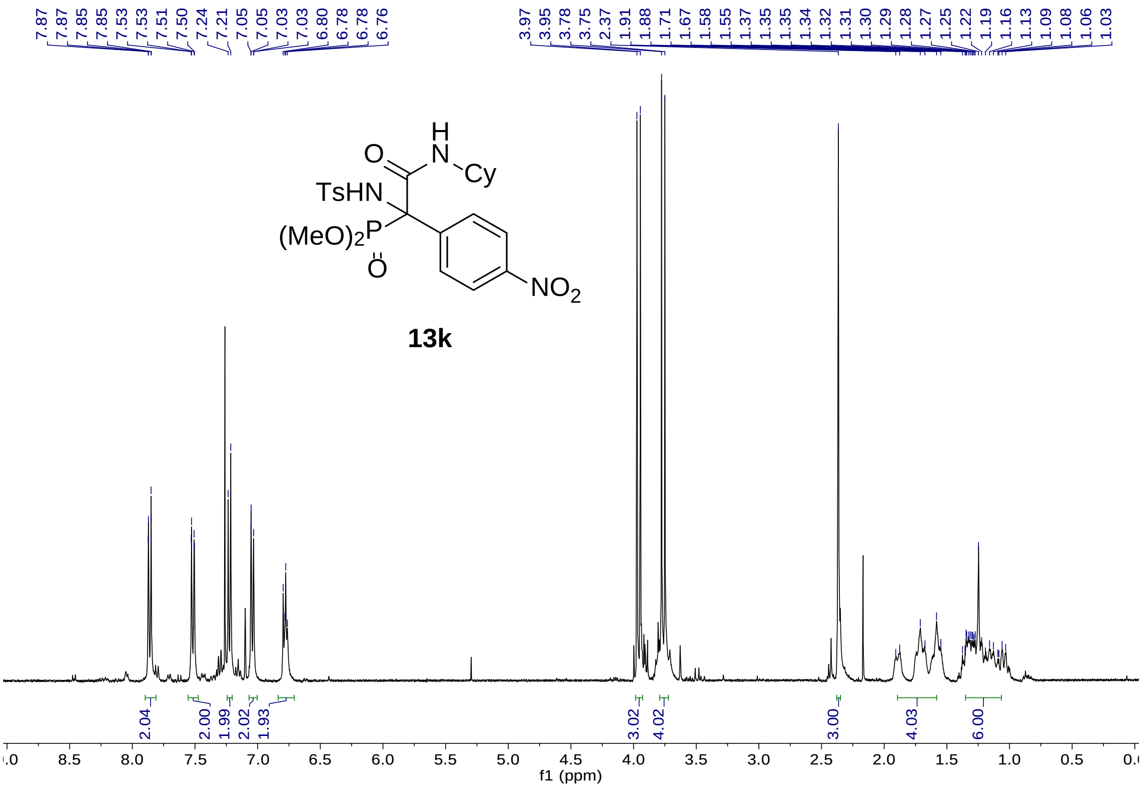

^{13}C NMR {1H} (101 MHz, $CDCl_3$)

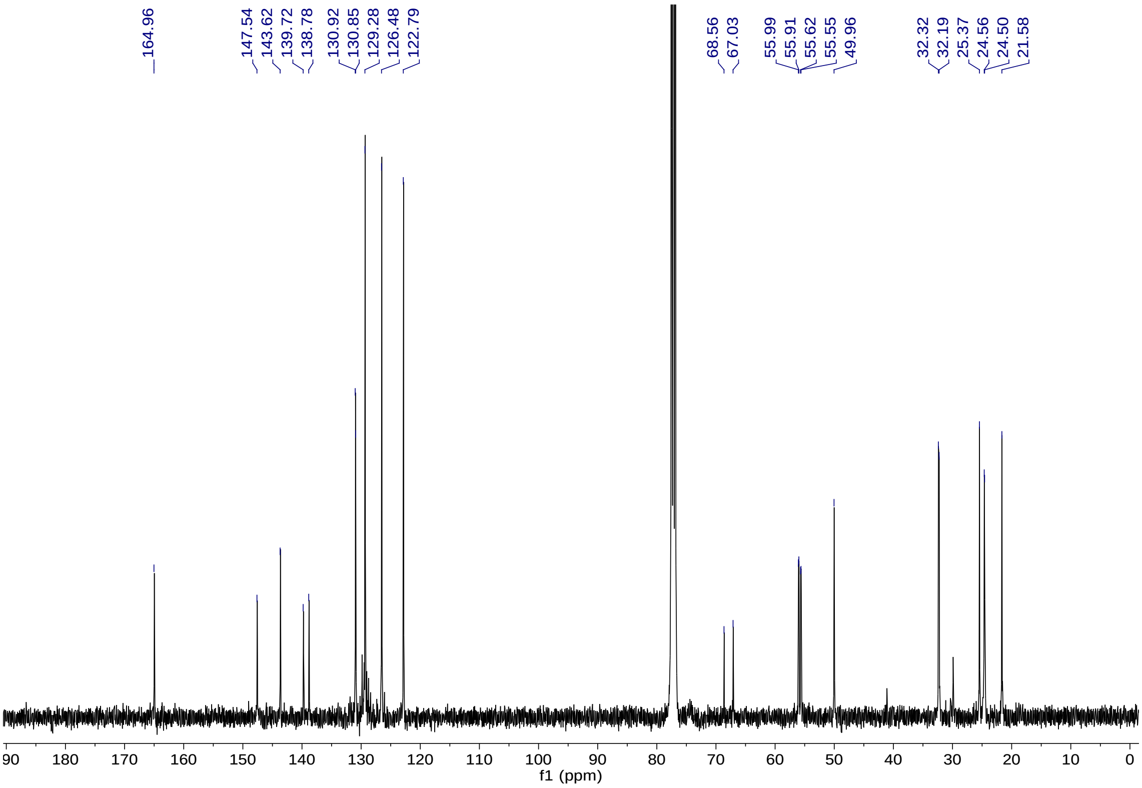

^{31}P NMR (121 MHz, $CDCl_3$)

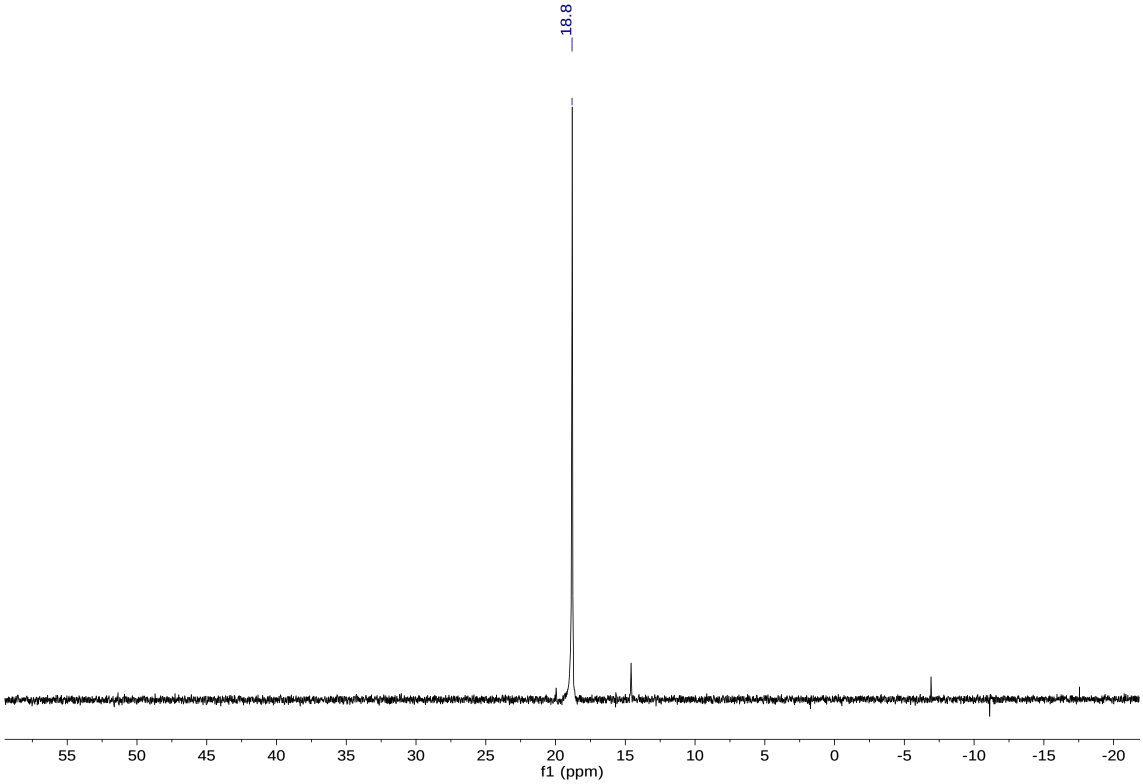

Dimethyl (1-(4-bromophenyl)-2-(cyclohexylamino)-1-((4-methylphenyl)sulfonamido)-2-oxoethyl)phosphonate (13l)

^{1}H NMR (400 MHz, $CDCl_3$)

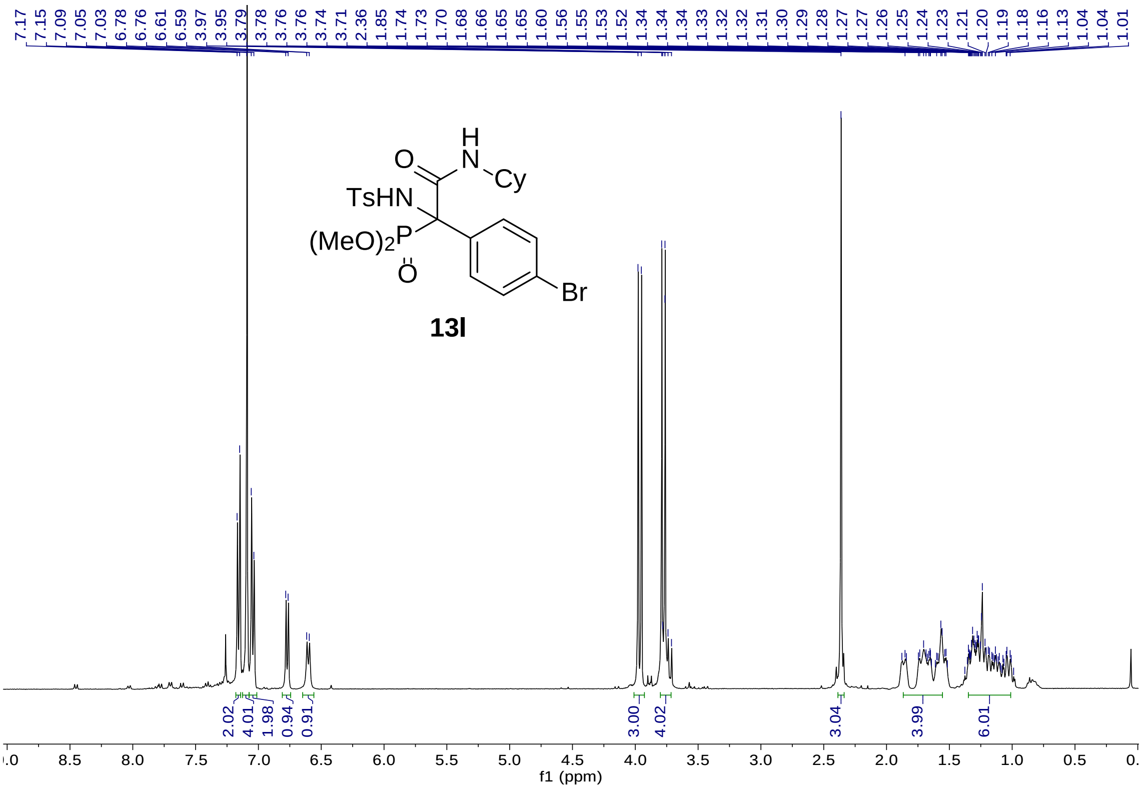

^{13}C NMR {1H} (101 MHz, $CDCl_3$)

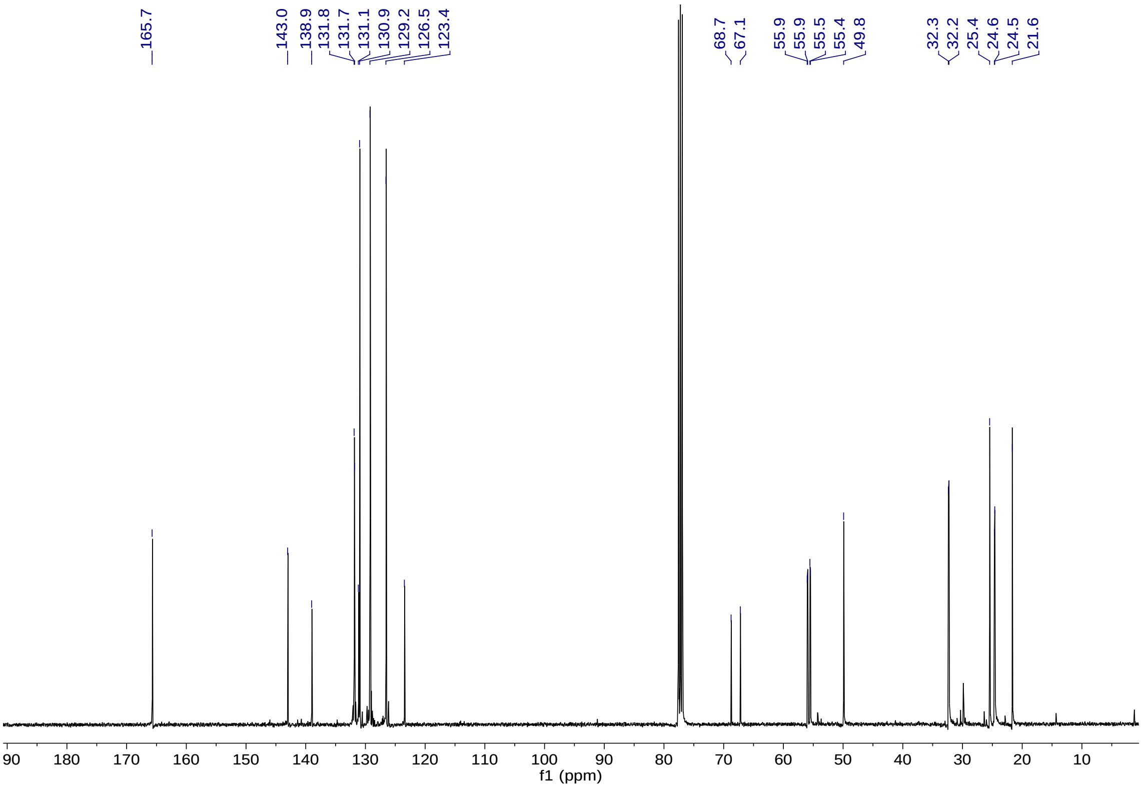

^{31}P NMR (121 MHz, $CDCl_3$)

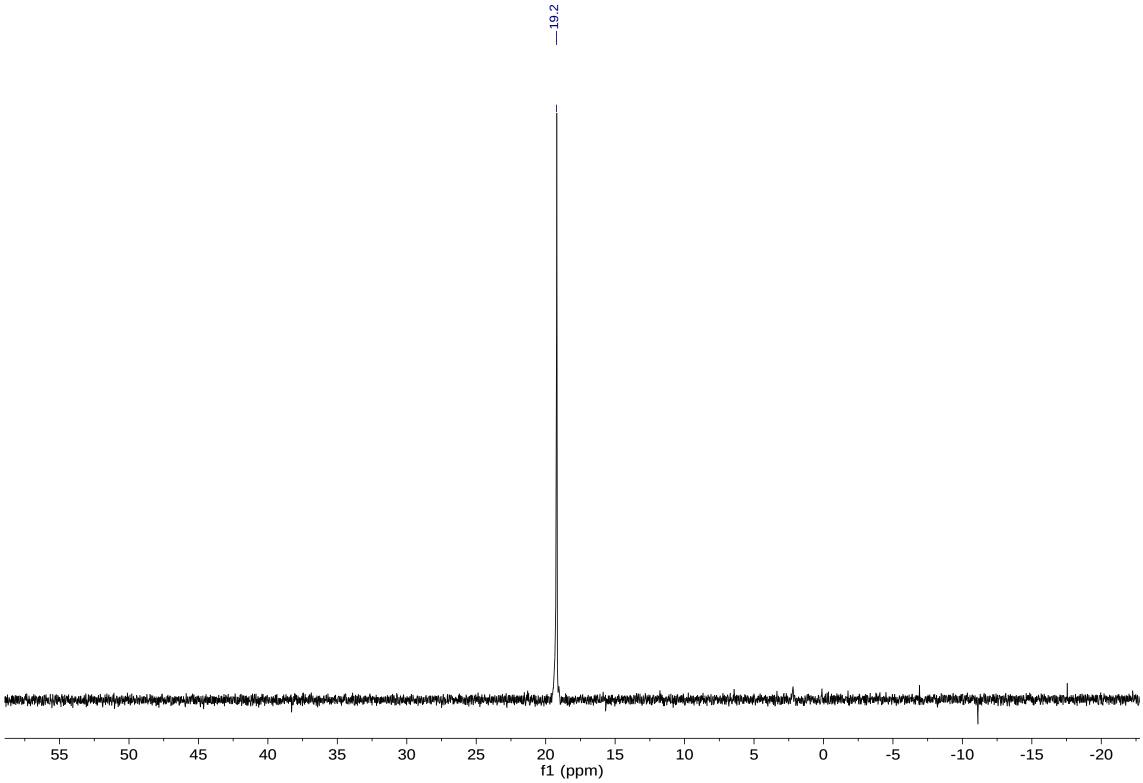

Dimethyl (1-(4-chlorophenyl)-2-(cyclohexylamino)-1-((4-methylphenyl)sulfonamido)-2-oxoethyl)phosphonate (13m)

^{1}H NMR (400 MHz, $CDCl_3$)

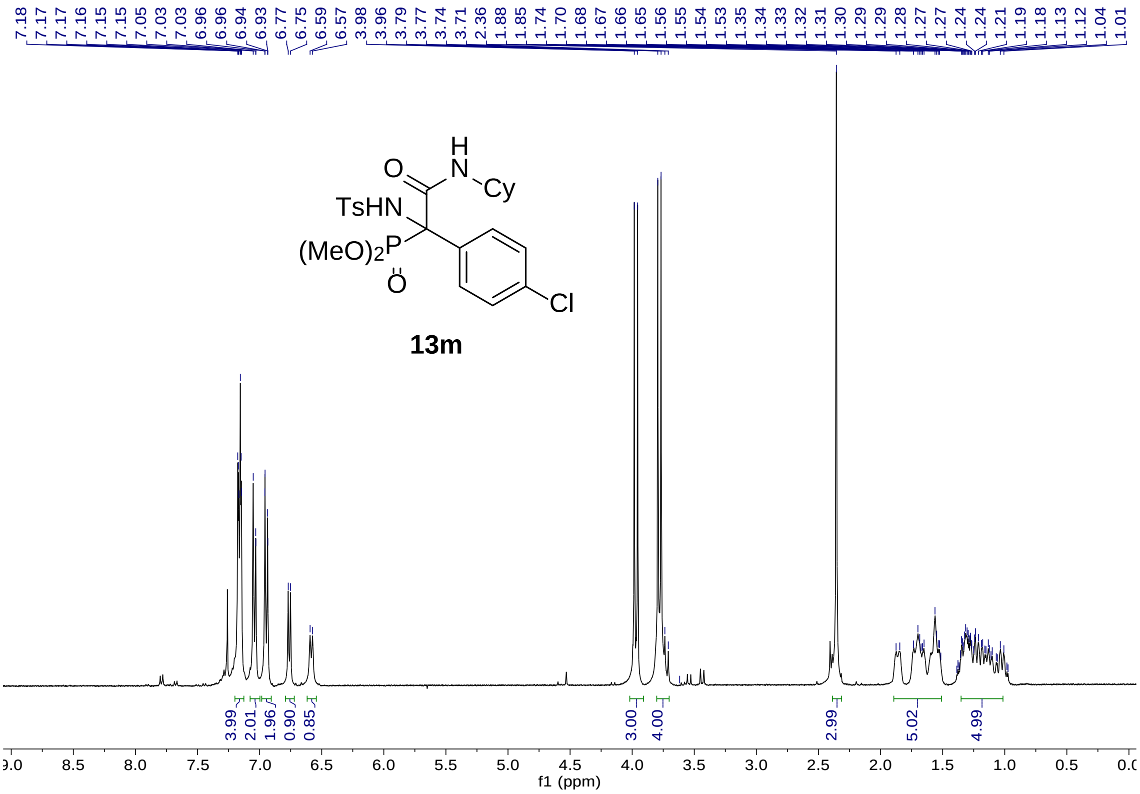

^{13}C NMR {1H} (101 MHz, $CDCl_3$)

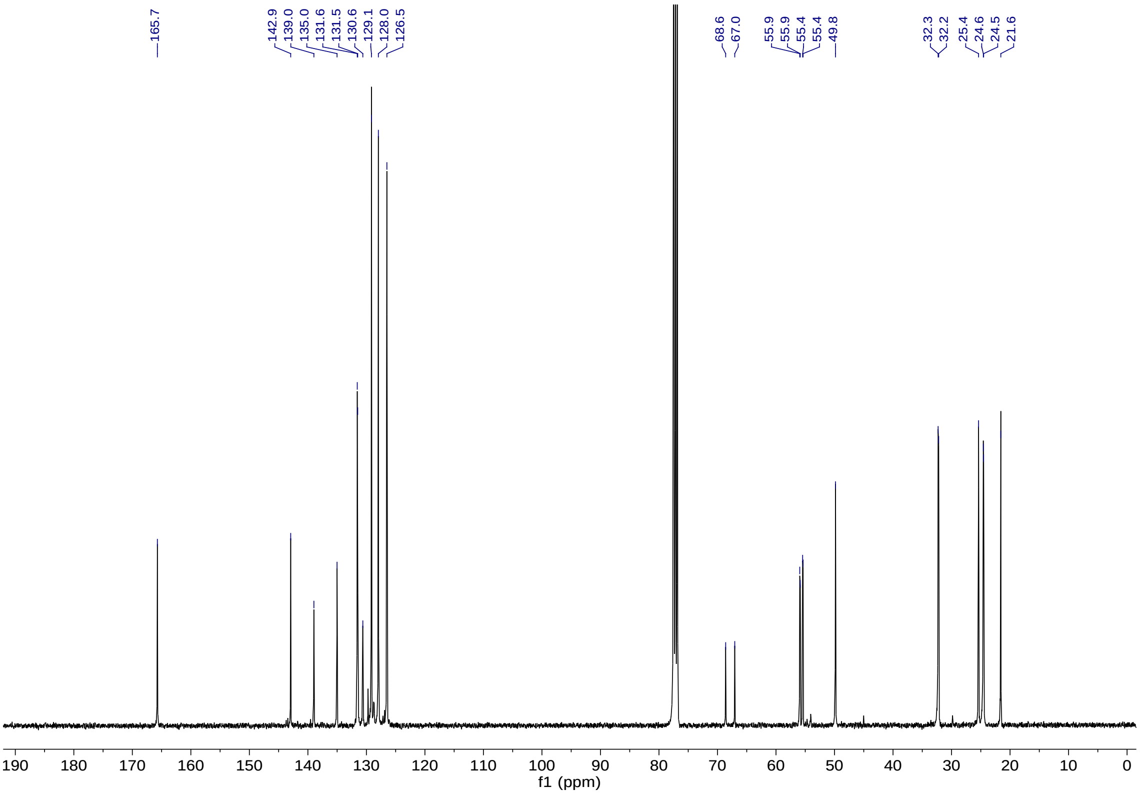

^{31}P NMR (121 MHz, CDCl$_3$)

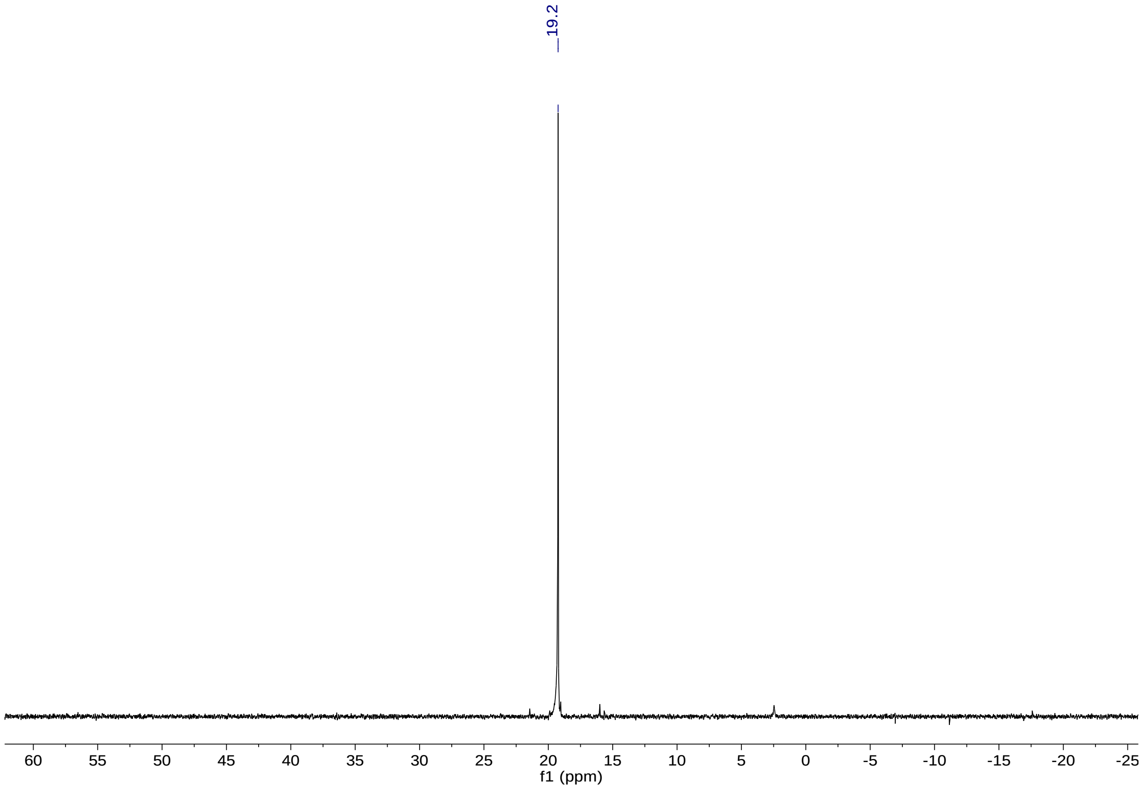

Dimethyl (2-(cyclohexylamino)-1-(2-fluorophenyl)-1-((4-methylphenyl)sulfonamido)-2-oxoethyl)phosphonate (13n)

^{1}H NMR (400 MHz, $CDCl_3$)

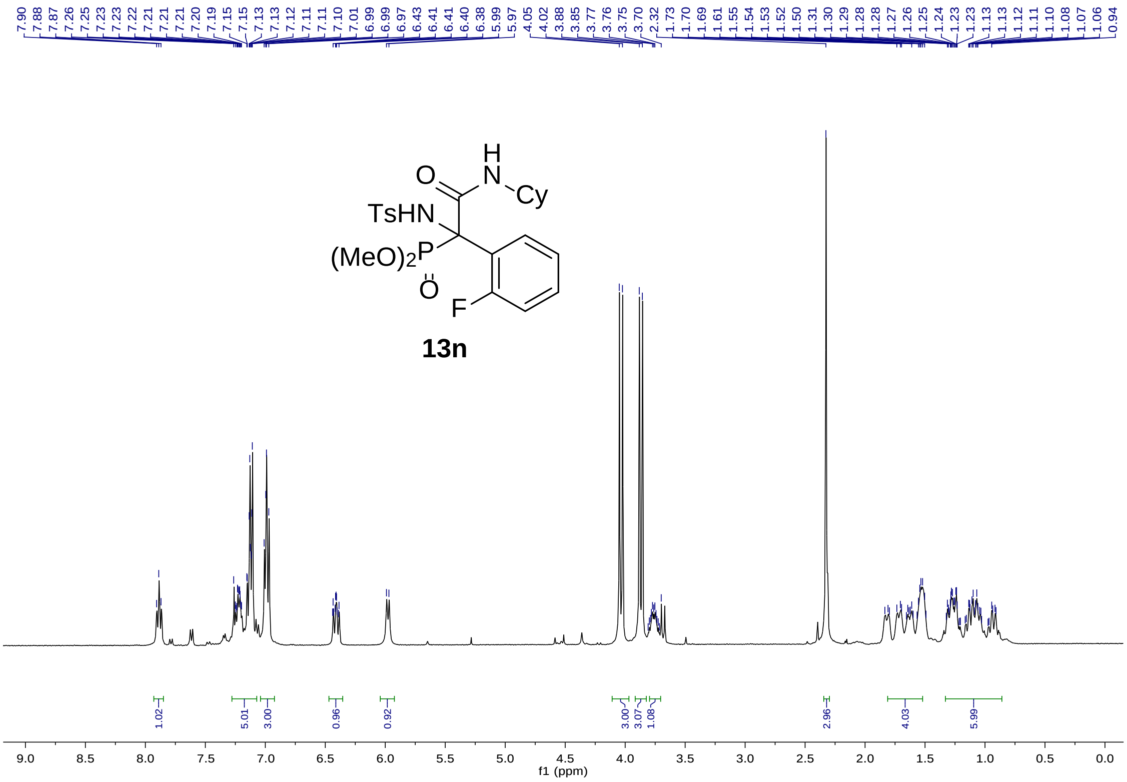

^{13}C NMR {1H} (101 MHz, $CDCl_3$)

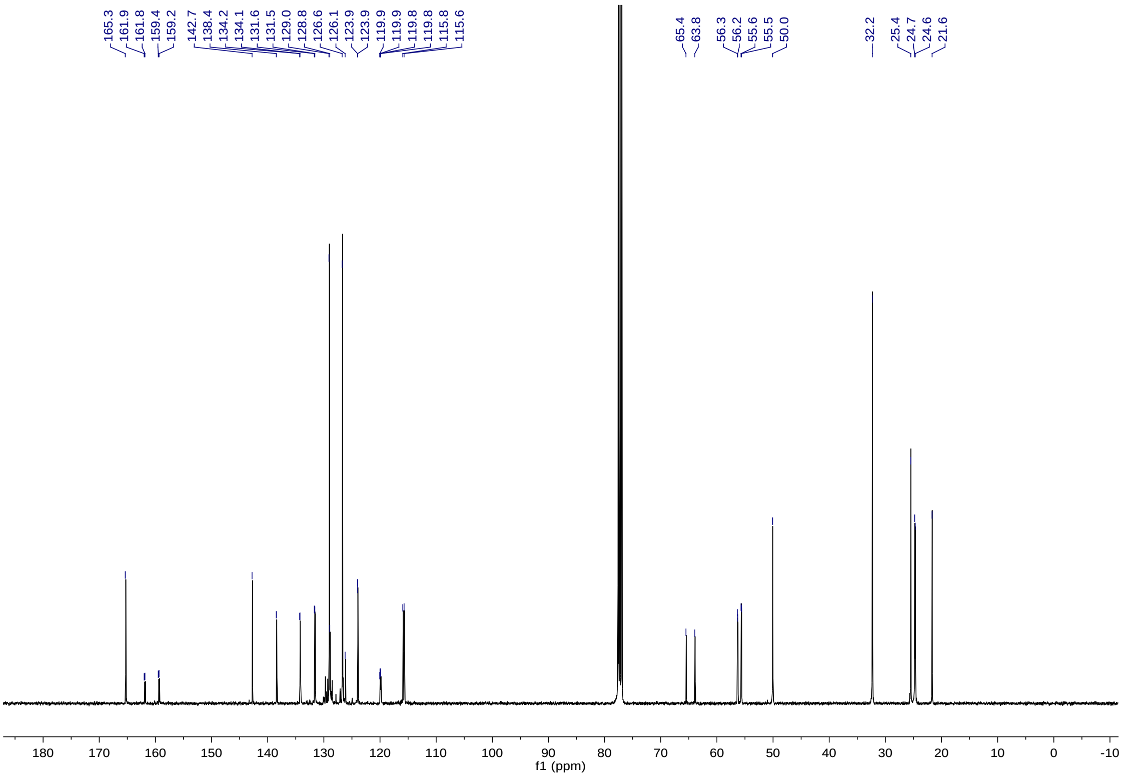

^{31}P NMR (121 MHz, $CDCl_3$)

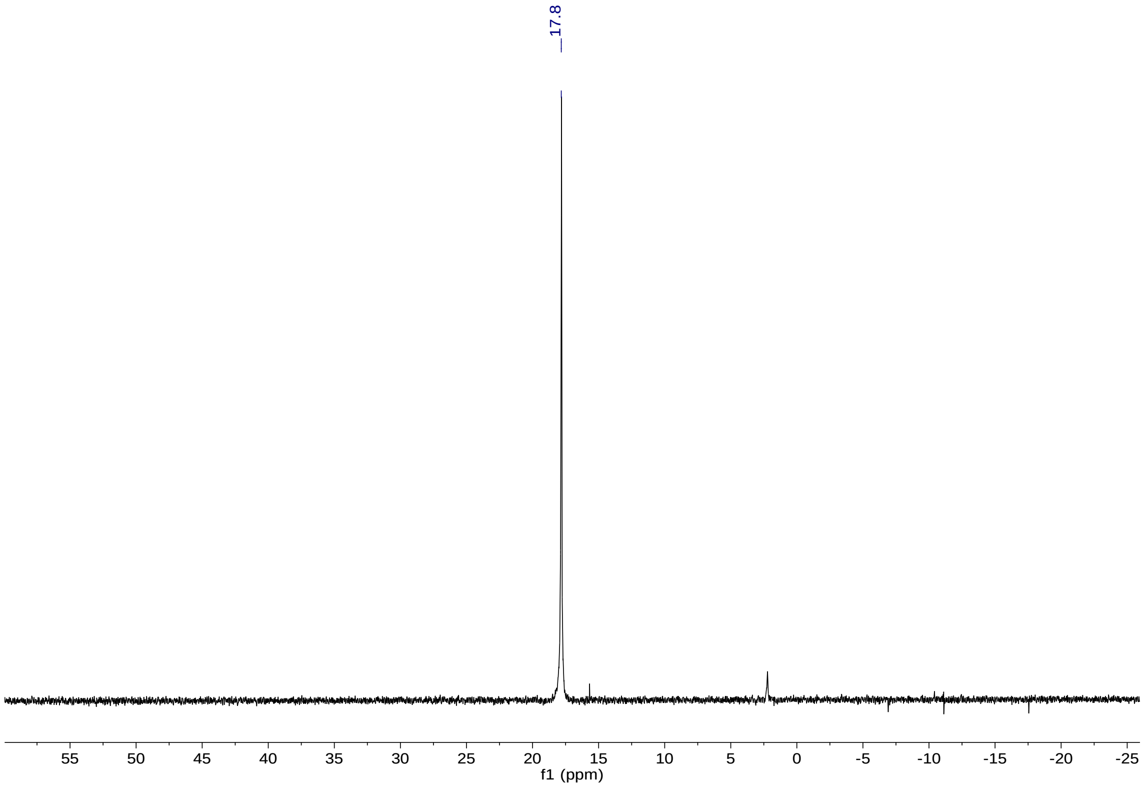

^{19}F NMR (282 MHz, $CDCl_3$)

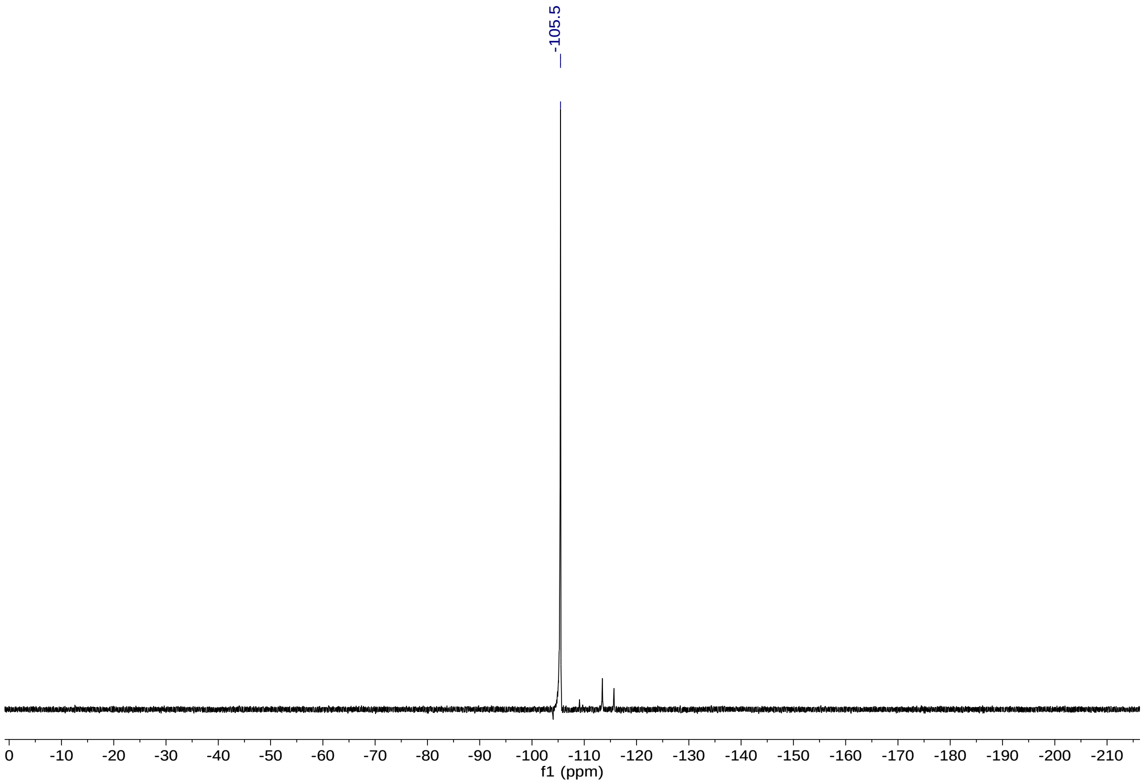

Dimethyl (2-(cyclohexylamino)-1-((4-methylphenyl)sulfonamido)-2-oxo-1-(perfluorophenyl)ethyl)phosphonate (13o)

[1]**H NMR** (400 MHz, $CDCl_3$)

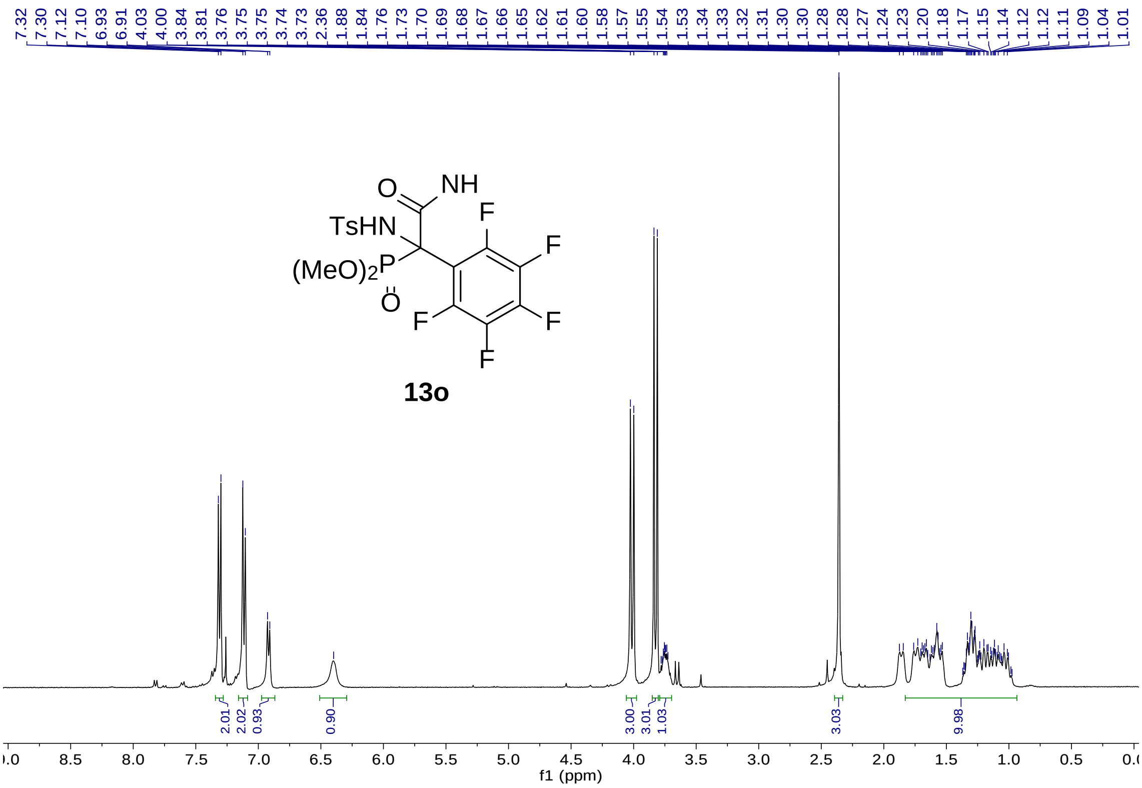

[13]**C NMR** {1H} (101 MHz, $CDCl_3$)

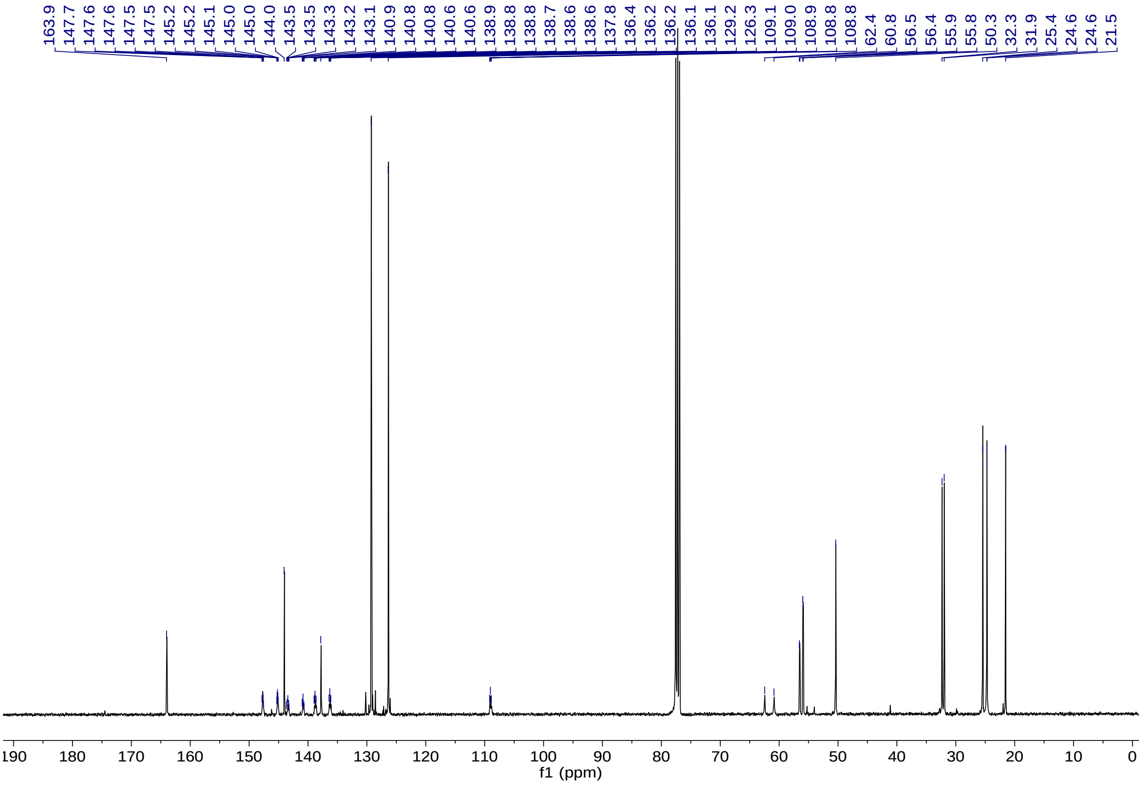

^{31}P NMR (121 MHz, $CDCl_3$)

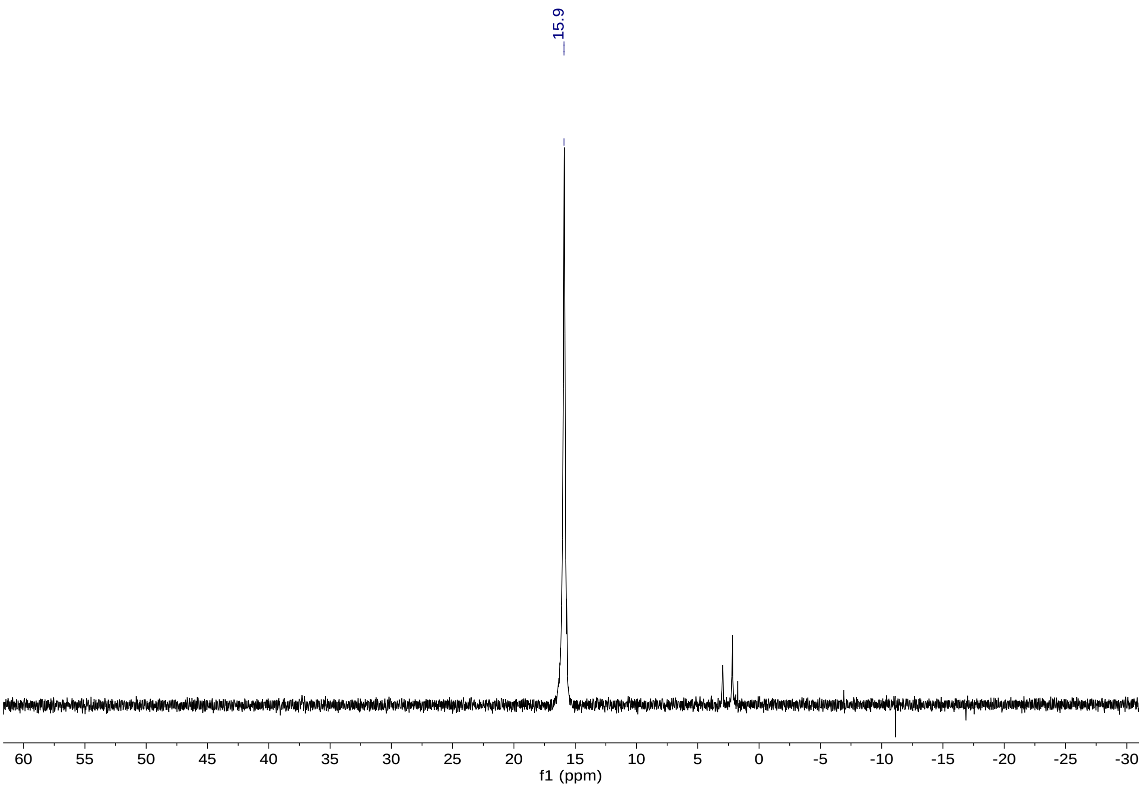

^{19}F NMR (282 MHz, $CDCl_3$)

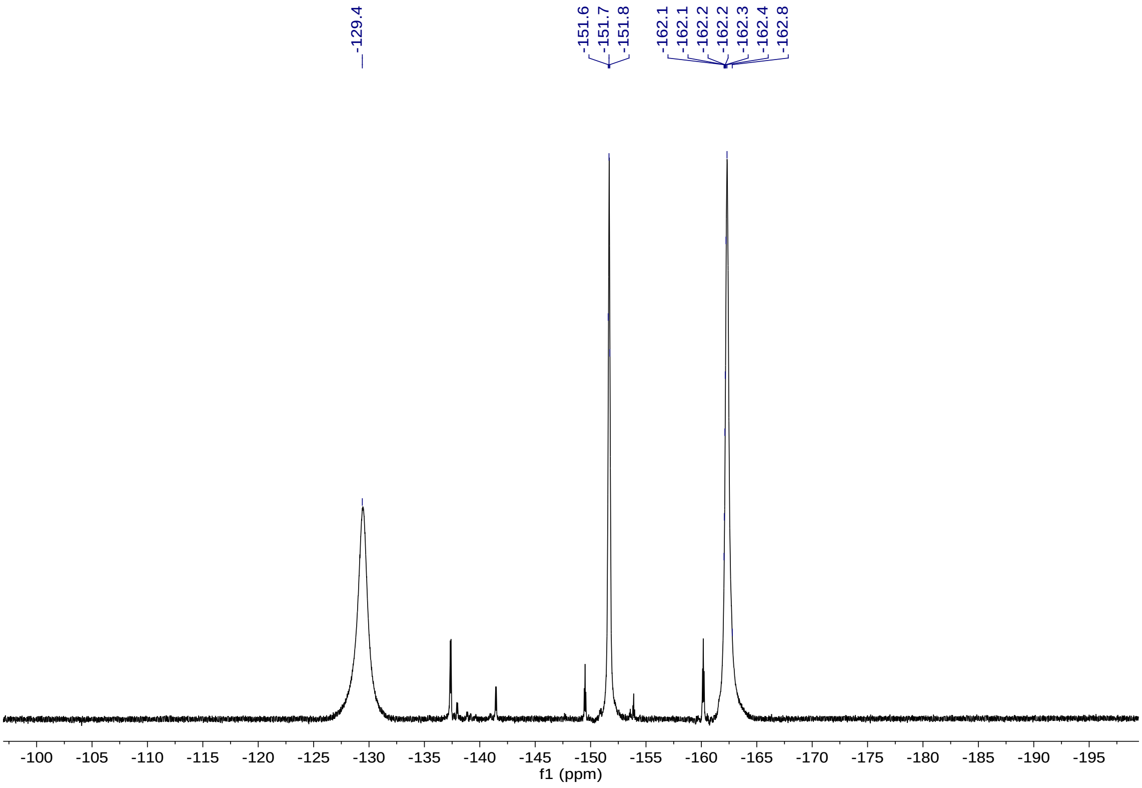

Dimethyl (2-(cyclohexylamino)-1-((4-methylphenyl)sulfonamido)-2-oxo-1-(4-((trichloromethyl)thio)phenyl)ethyl)phosphonate (13p)

^{1}H NMR (400 MHz, $CDCl_3$)

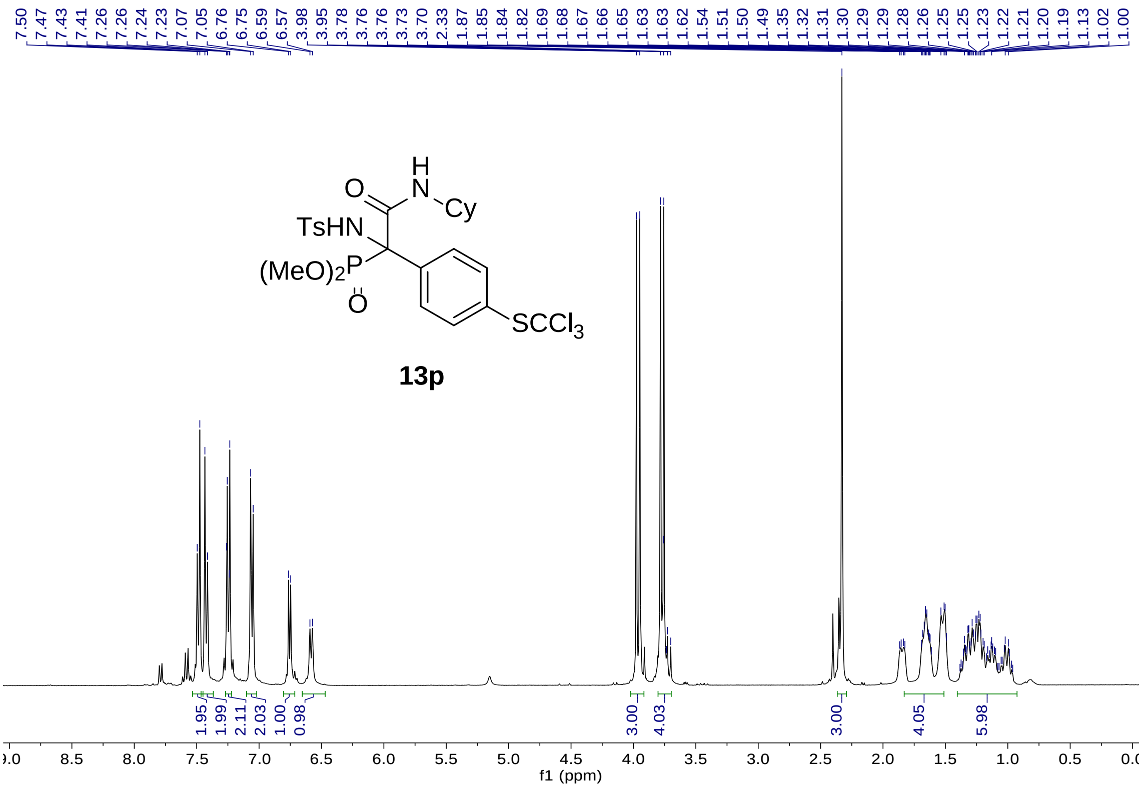

^{13}C NMR {1H} (101 MHz, $CDCl_3$)

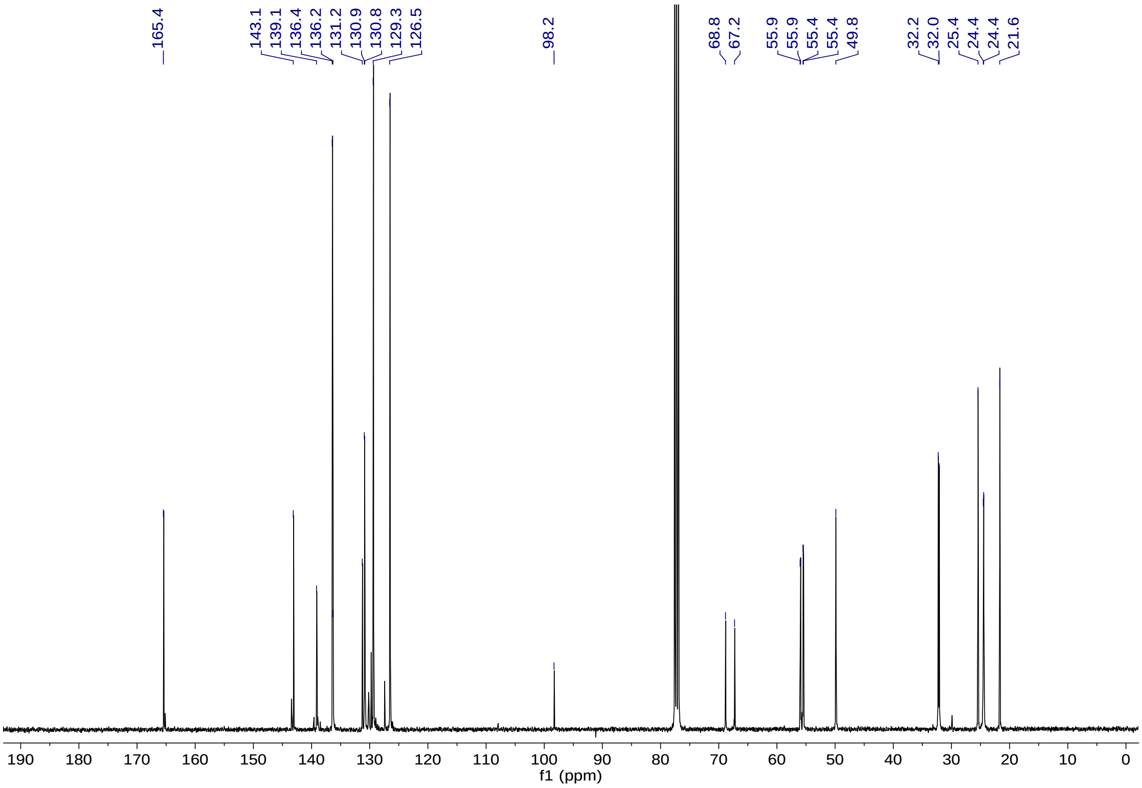

^{31}P NMR (121 MHz, $CDCl_3$)

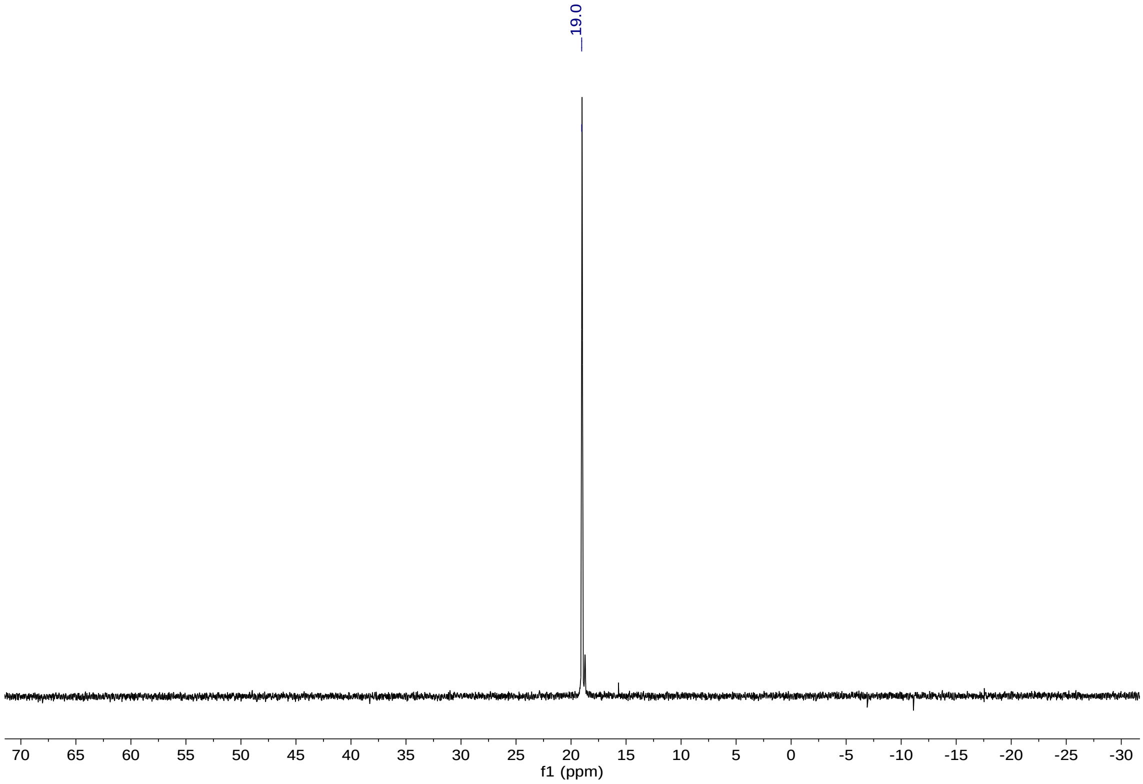

Dimethyl (1-(3-chloro-4-methoxyphenyl)-2-(cyclohexylamino)-1-((4-methylphenyl)-sulfonamido)-2-oxoethyl)phosphonate (13q).

^{1}H NMR (400 MHz, $CDCl_3$)

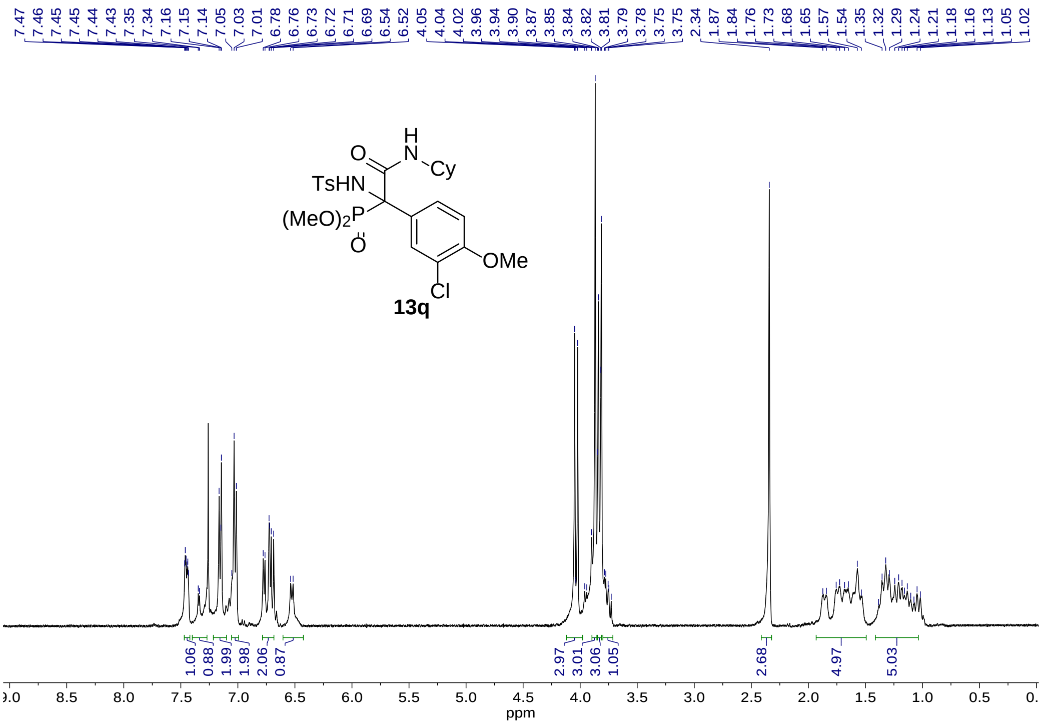

^{13}C NMR {^{1}H} (101 MHz, $CDCl_3$)

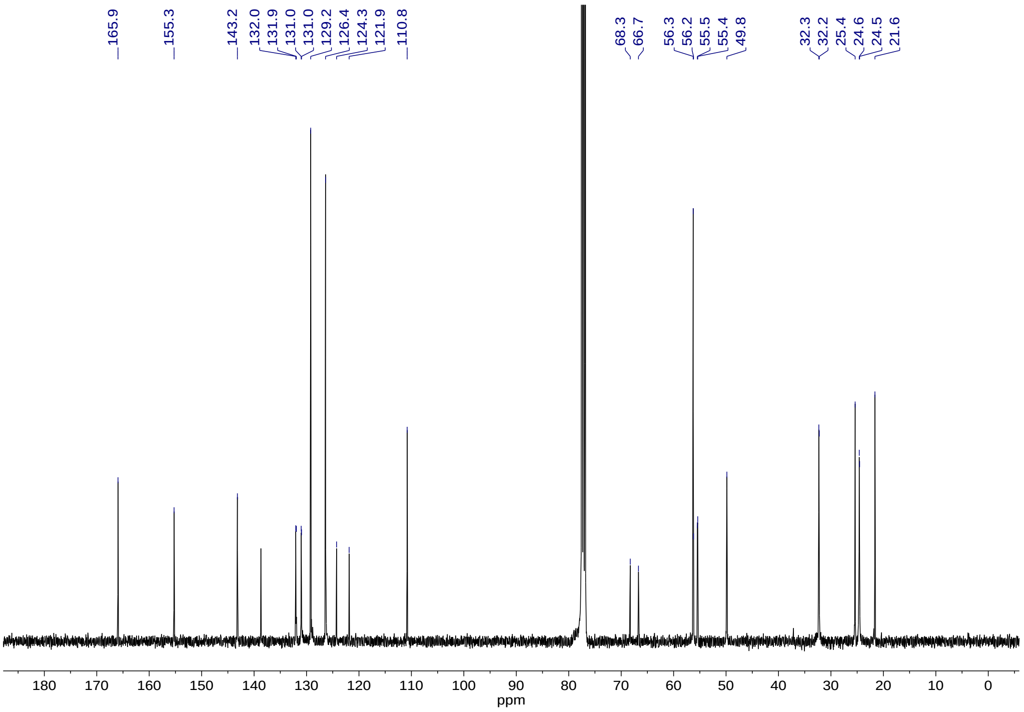

^{31}P NMR (121 MHz, $CDCl_3$)

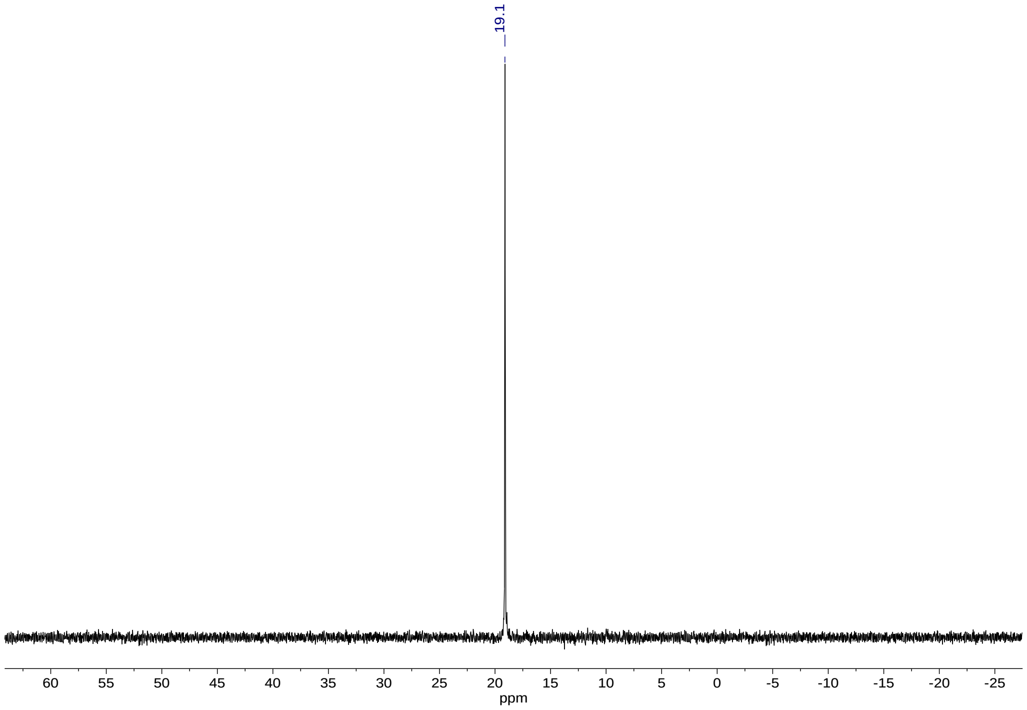

Dimethyl (2-(cyclohexylamino)-1-((4-methylphenyl)sulfonamido)-2-oxo-1-(4-(trifluoromethyl)phenyl)ethyl)phosphonate (13r)

^{1}H NMR (400 MHz, $CDCl_3$)

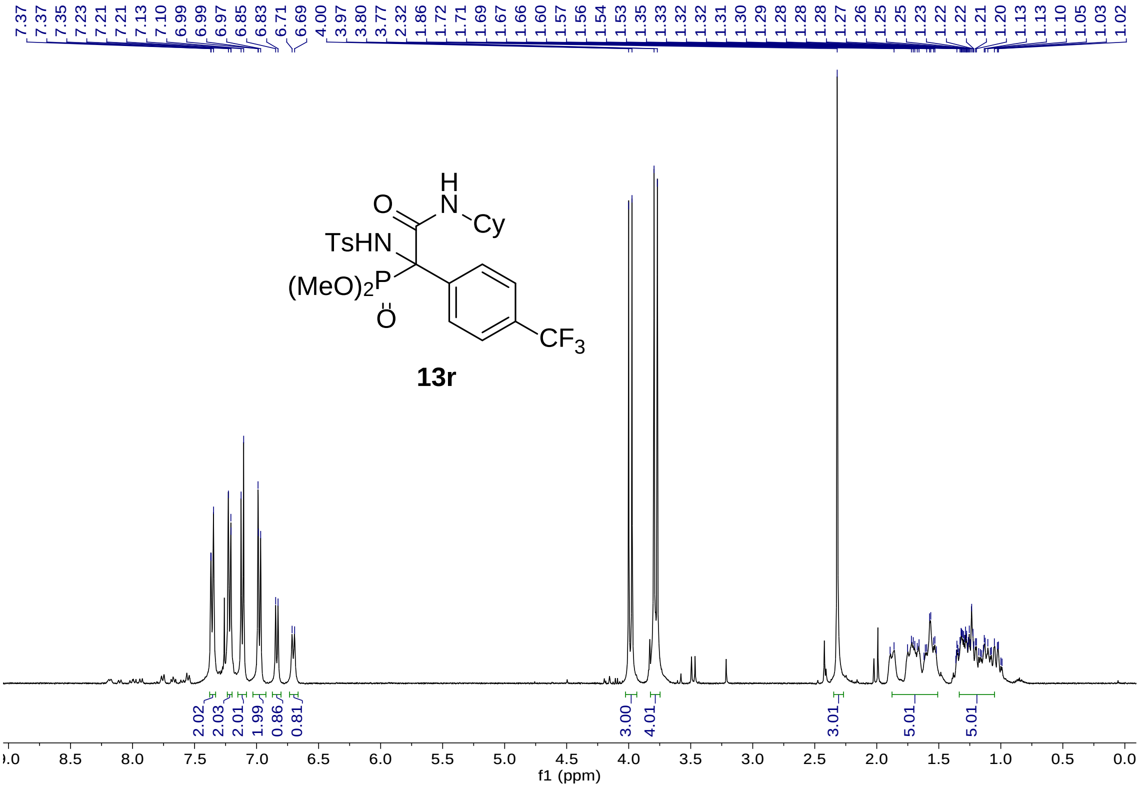

^{13}C NMR {1H} (101 MHz, $CDCl_3$)

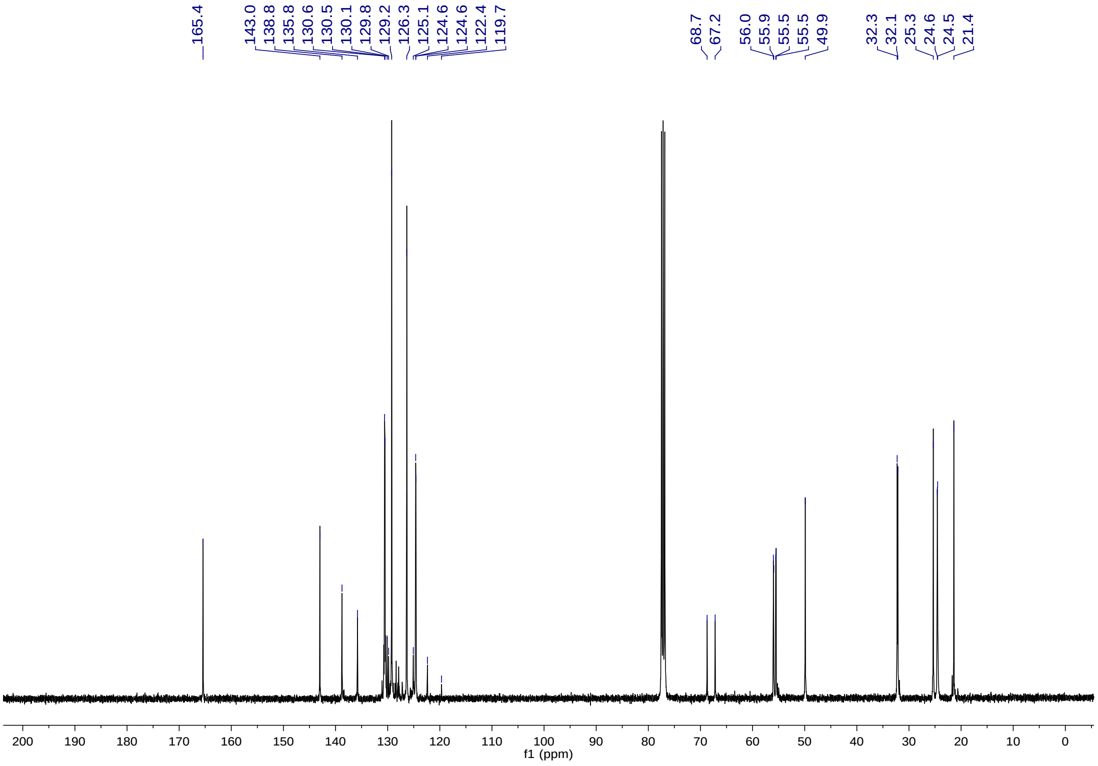

^{31}P NMR (121 MHz, $CDCl_3$)

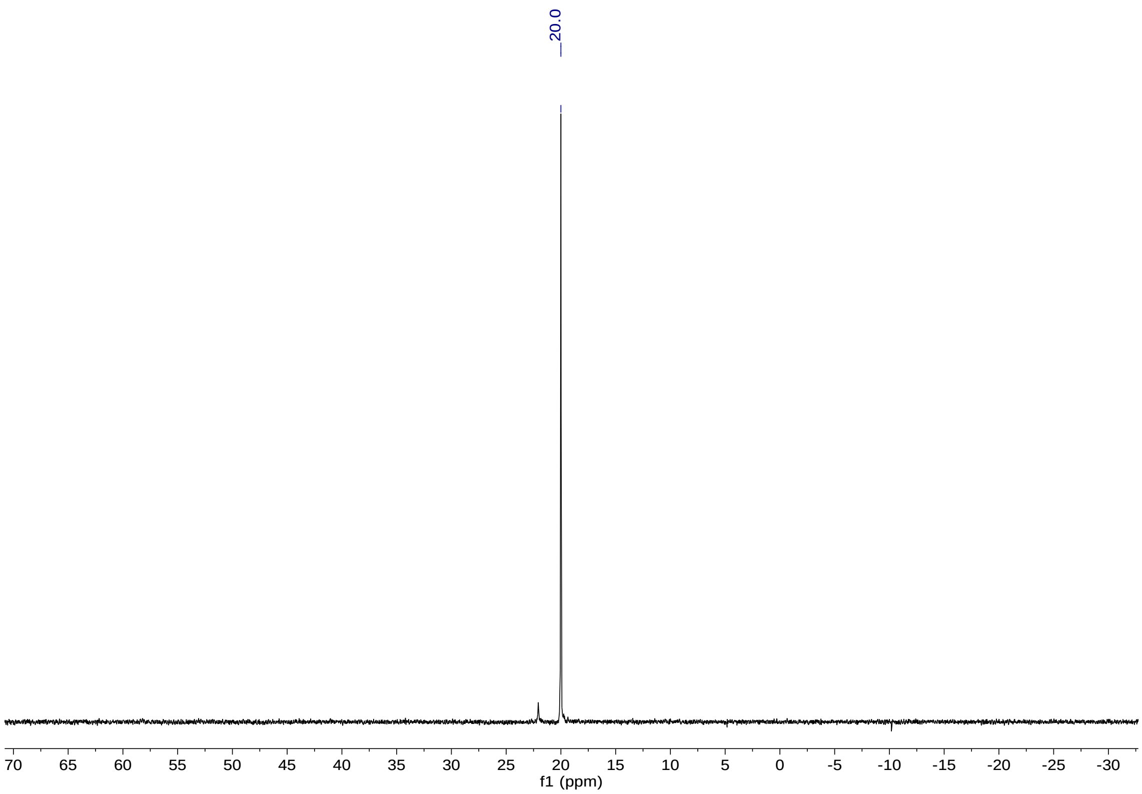

^{19}F NMR (282 MHz, $CDCl_3$)

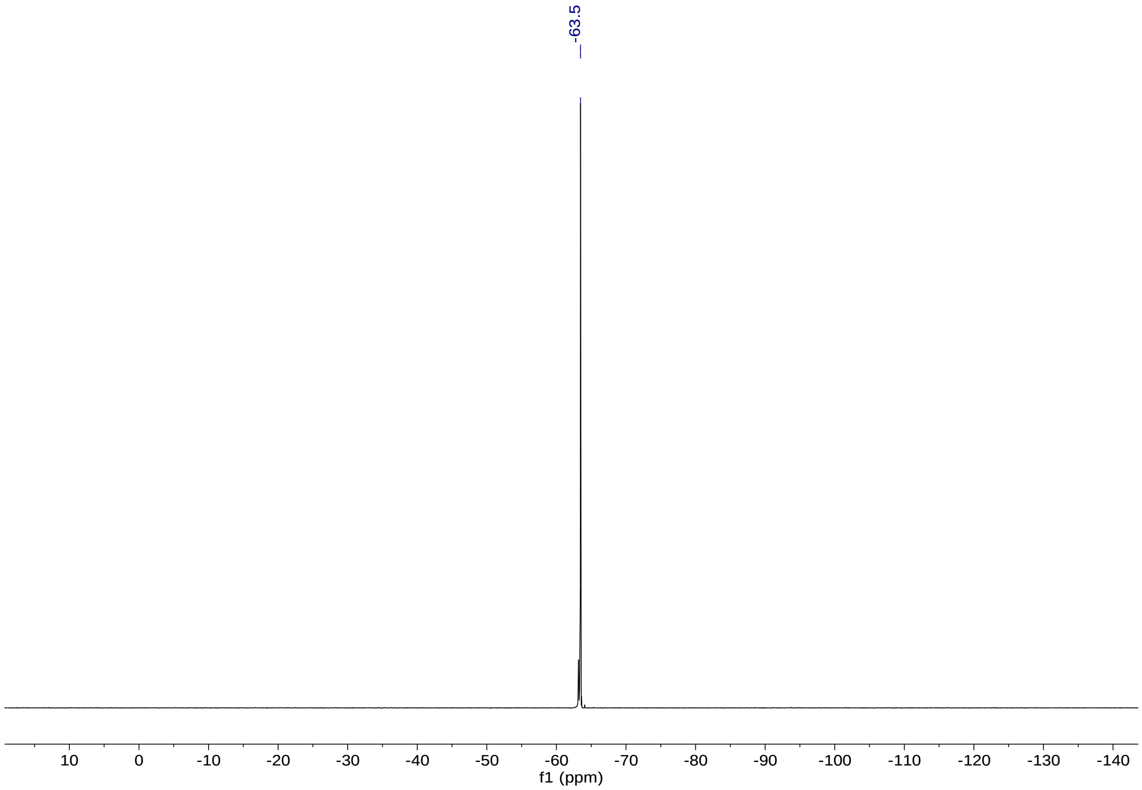

Dimethyl (2-(cyclohexylamino)-2-oxo-1-(2-phenylacetamido)ethyl)phosphonate (16).

^{1}H NMR (300 MHz, $CDCl_3$)

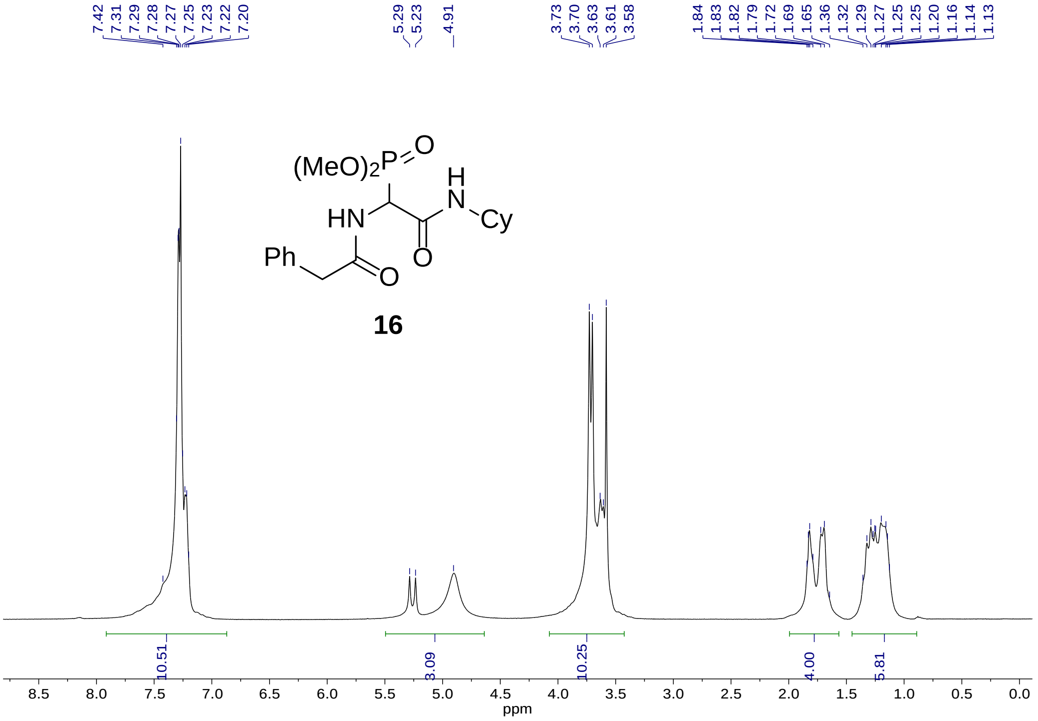

^{13}C NMR {^{1}H} (101 MHz, CD_3OD)

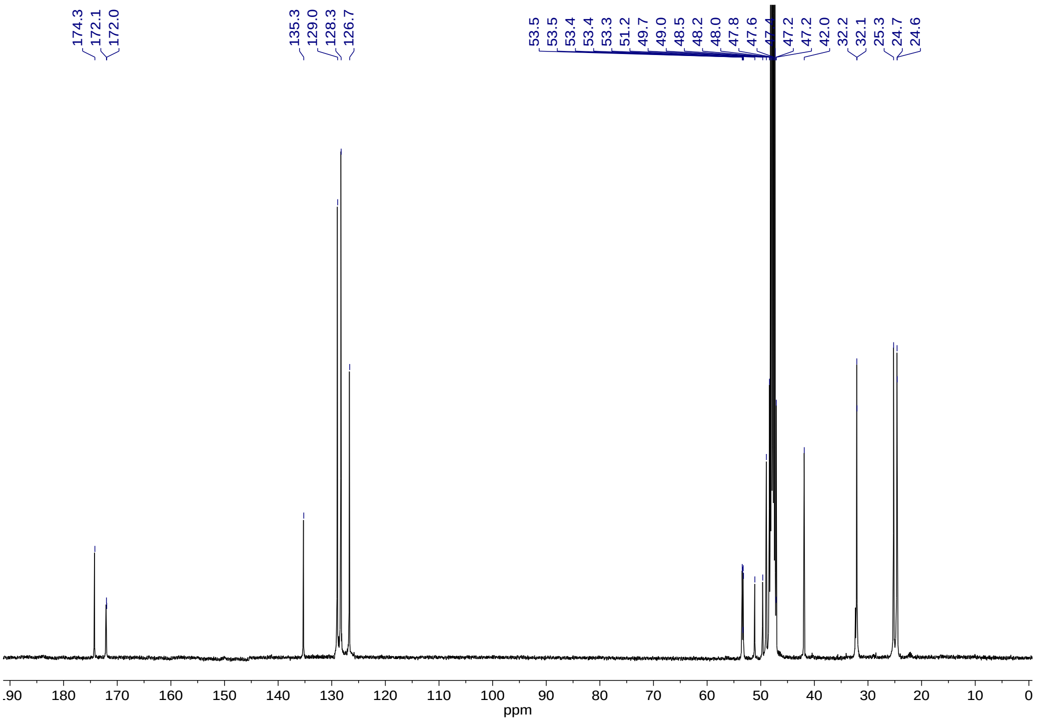

^{31}P NMR (121 MHz, $CDCl_3$)

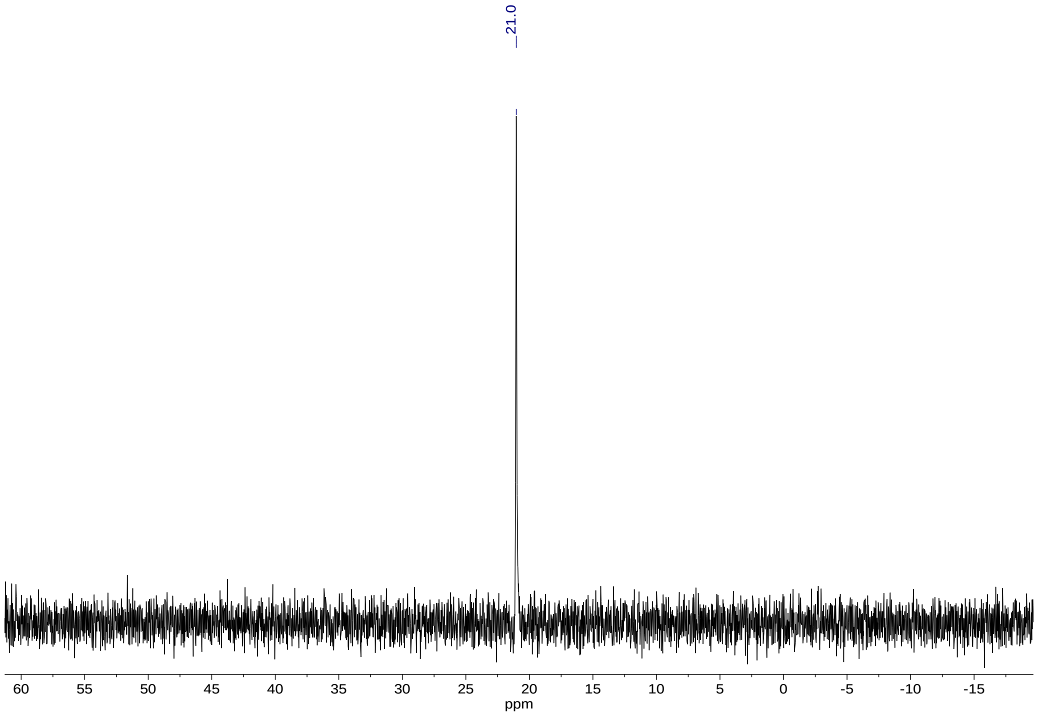

Dimethyl (2-(cyclohexylamino)-2-oxo-1-(2-phenyl-N-tosylacetamido)-1-(4-(trifluoromethyl)phenyl)ethyl)phosphonate (17a)

[1]**H NMR** (400 MHz, $CDCl_3$)

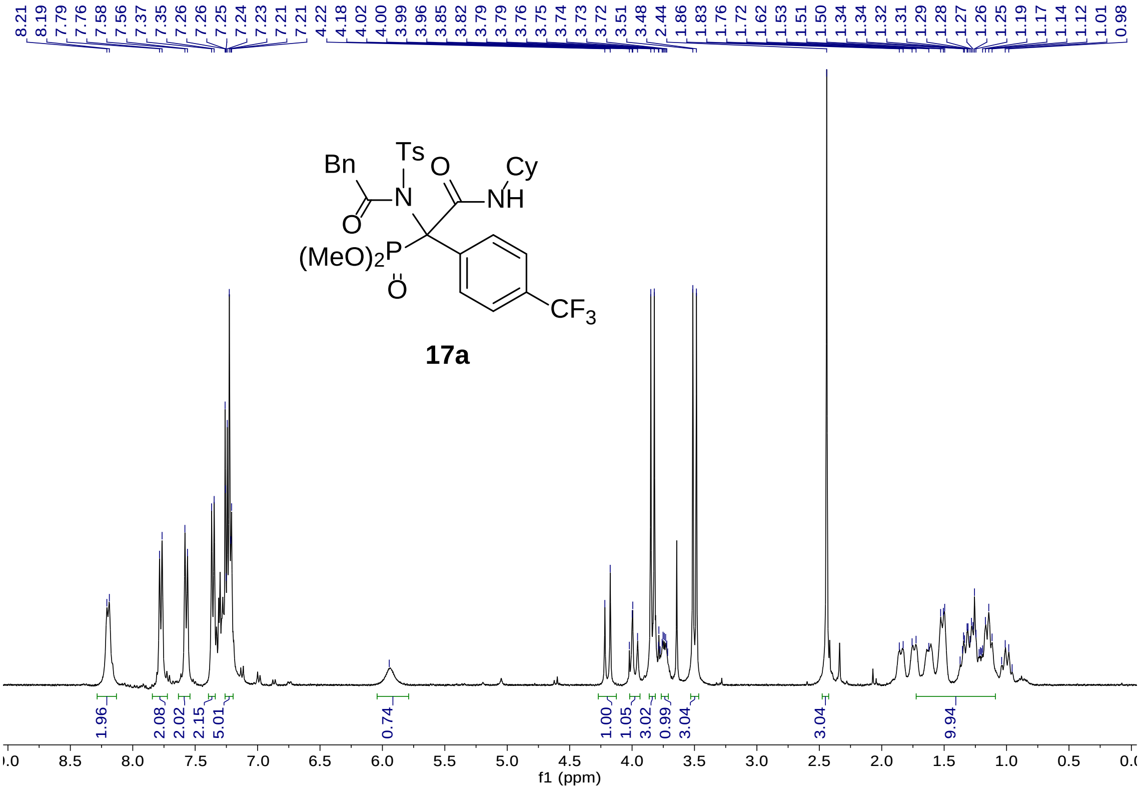

[13]**C NMR** {1H} (101 MHz, $CDCl_3$)

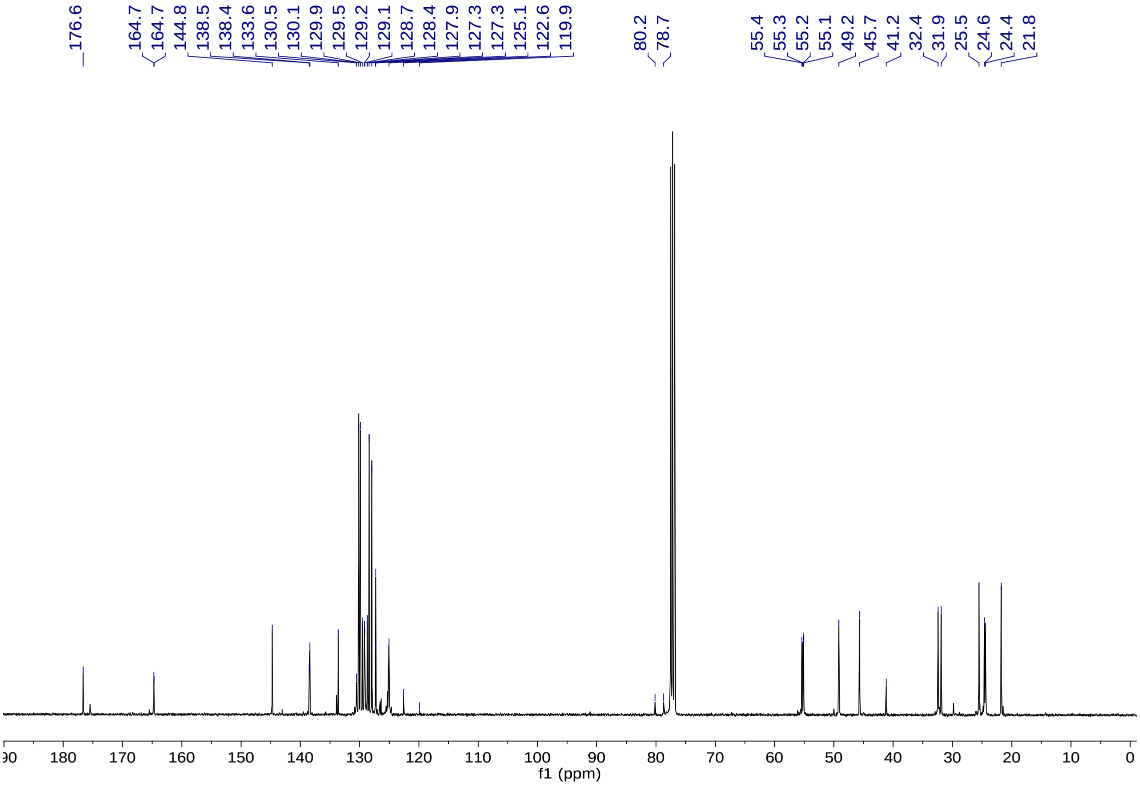

^{31}P NMR (121 MHz, $CDCl_3$)

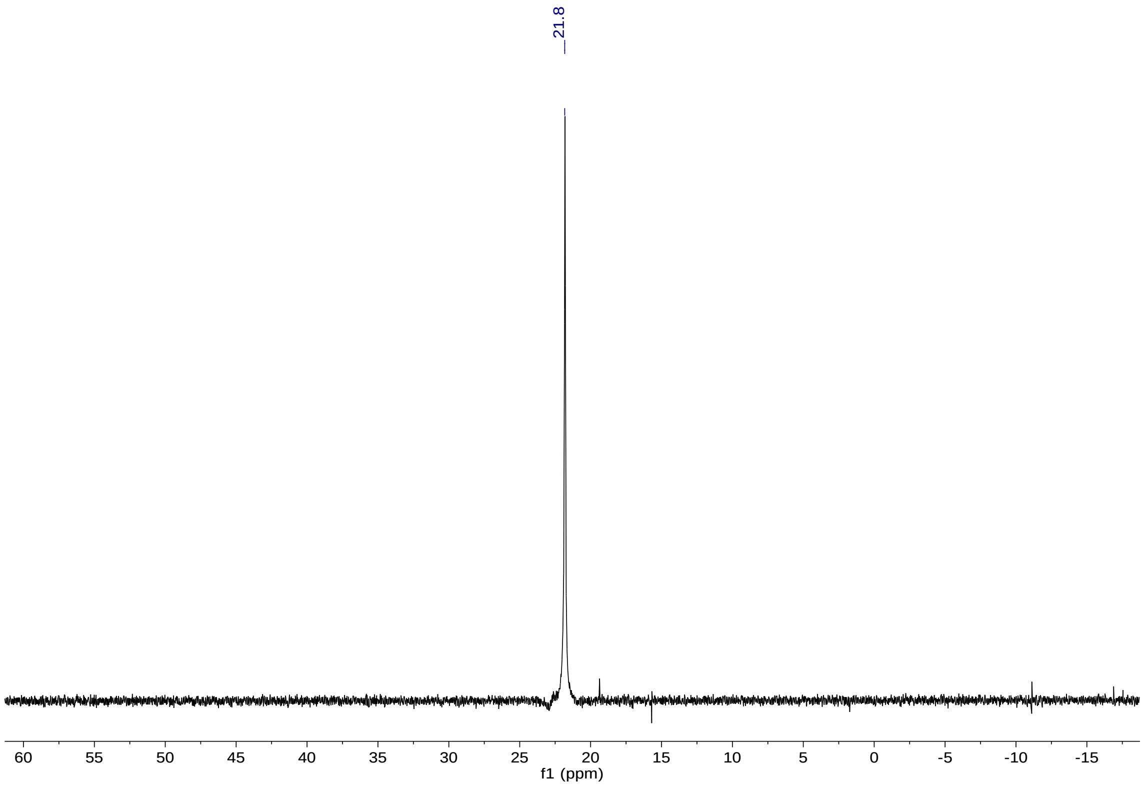

^{19}F NMR (282 MHz, $CDCl_3$)

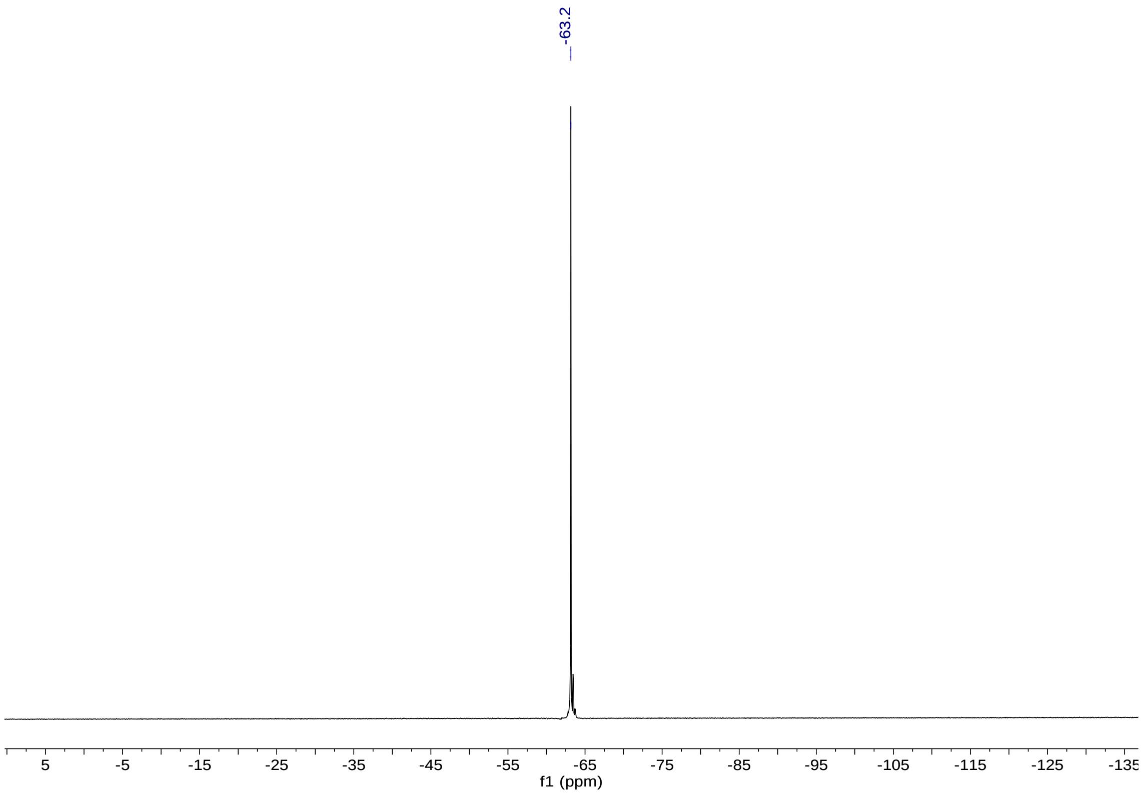

Dimethyl (2-(cyclohexylamino)-1-(4-fluorophenyl)-1-((4-methylphenyl)sulfonamido)-2-oxoethyl)phosphonate (17b).

^{1}H NMR (400 MHz, $CDCl_3$)

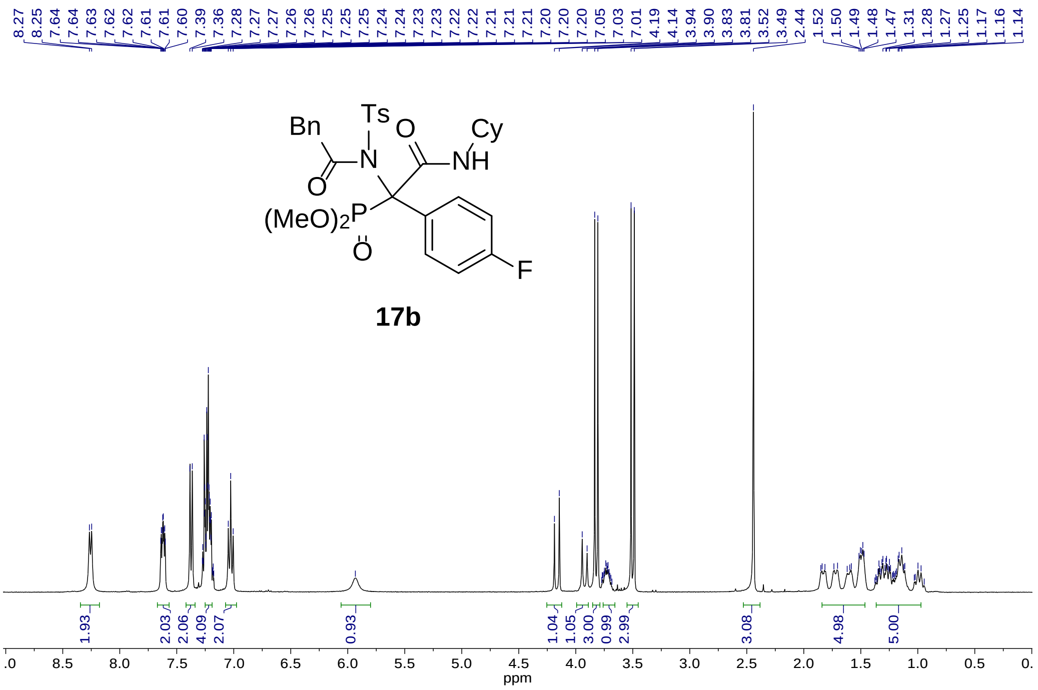

^{13}C {^{1}H} NMR (101 MHz, $CDCl_3$)

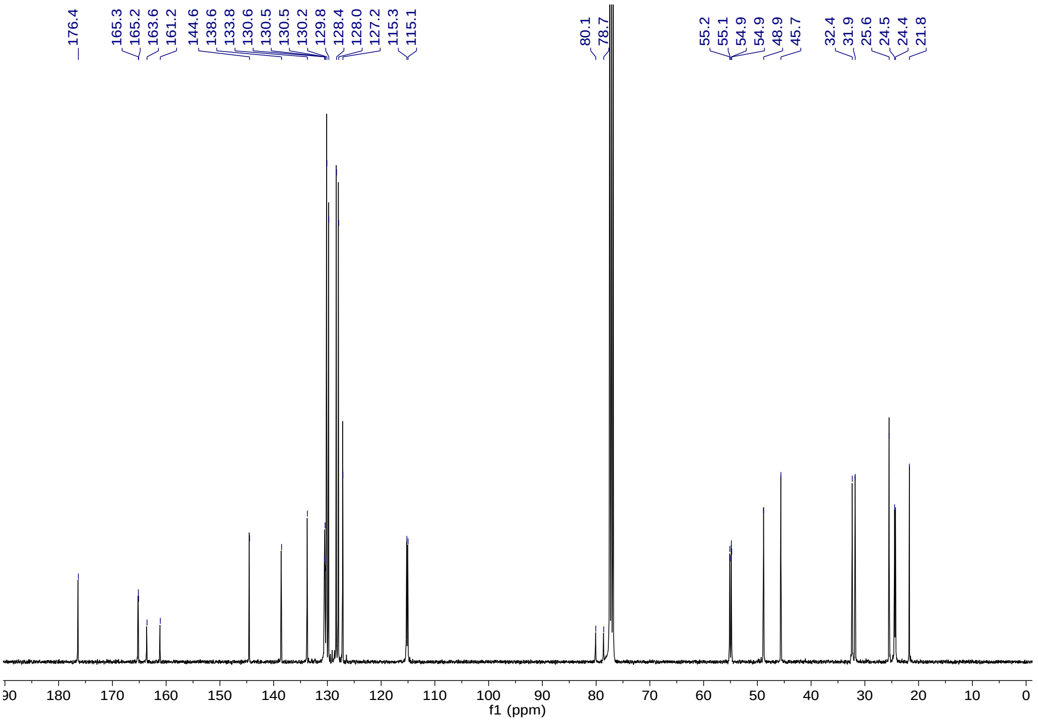

^{31}P NMR (121 MHz, $CDCl_3$)

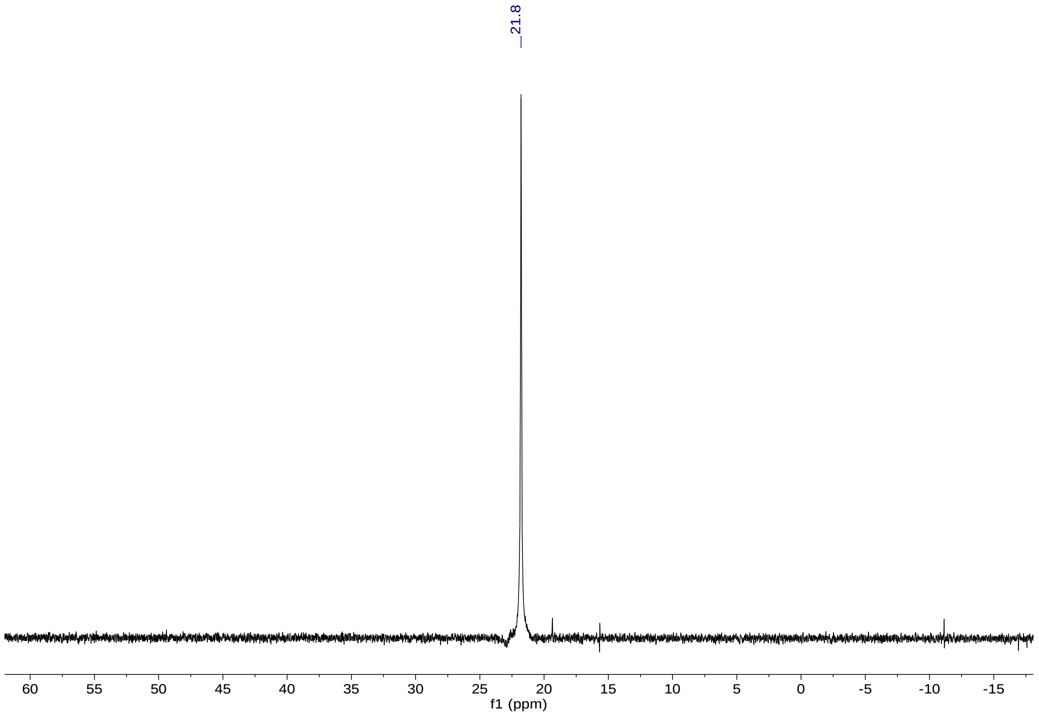

^{19}F NMR (282 MHz, $CDCl_3$)

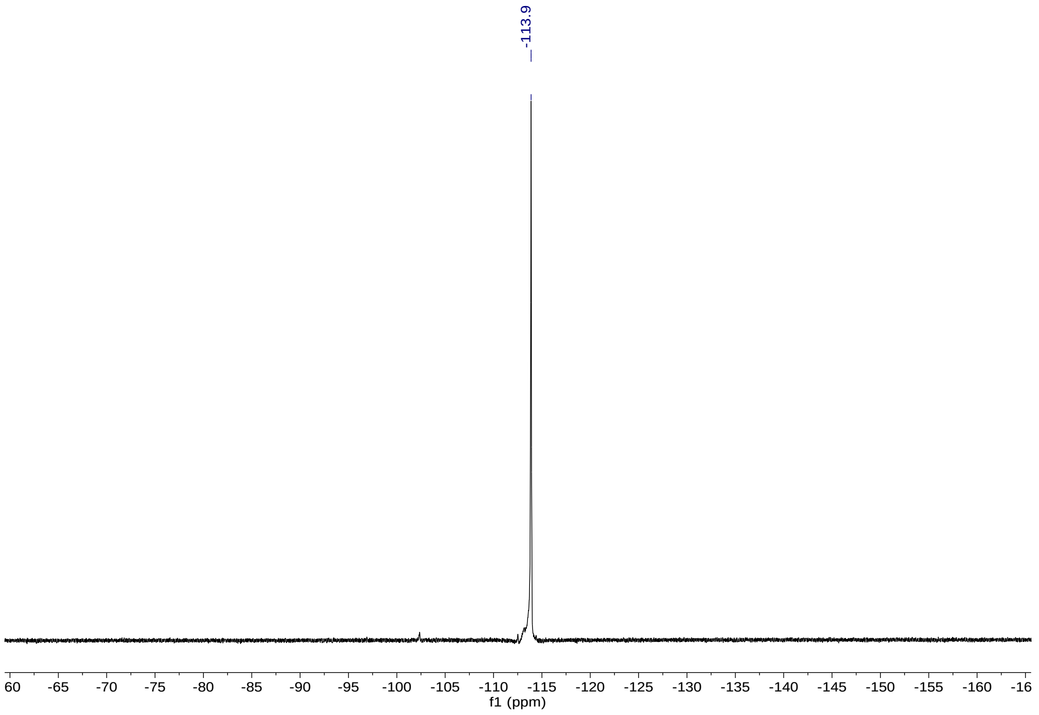

(2-((2-Methoxy-2-oxoethyl)amino)-1-((4-methylphenyl)sulfonamido)-2-oxo-1-phenylethyl)phosphonic acid (24)..

¹H NMR (400 MHz, D_2O)

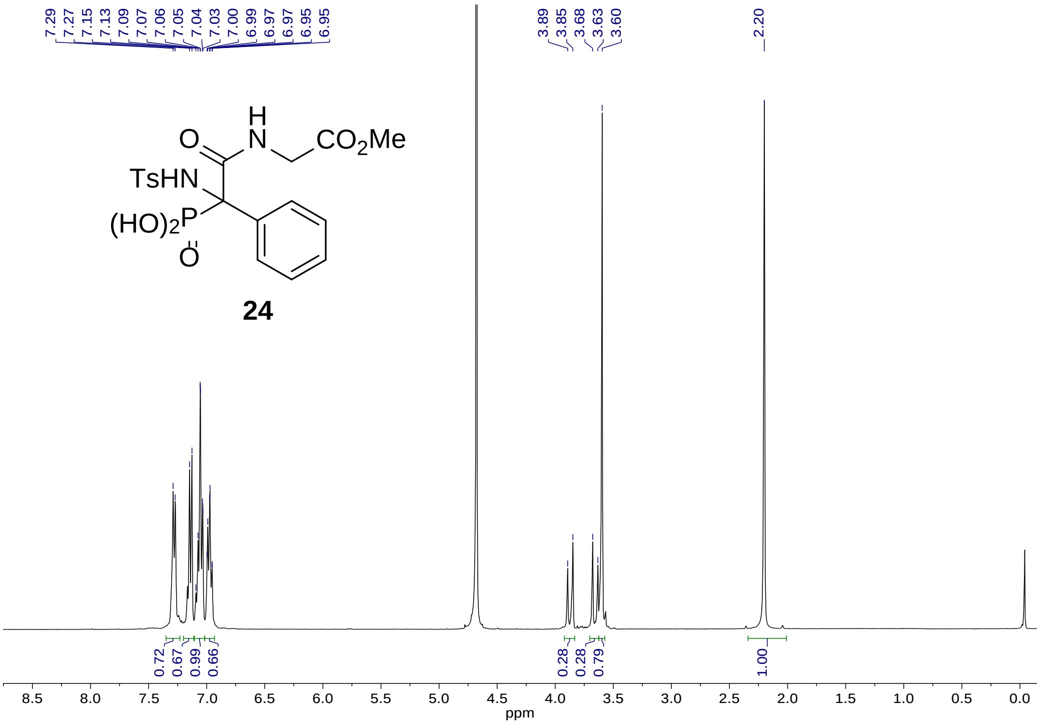

¹³C NMR {¹H} (101 MHz, D_2O)

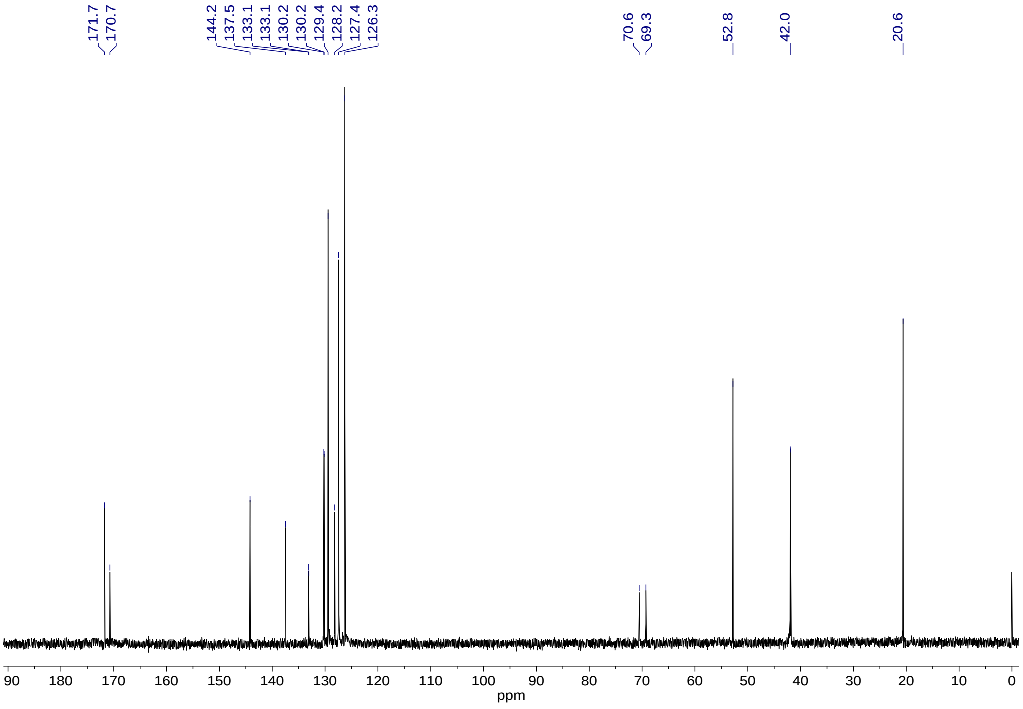

^{31}P NMR (121 MHz, D_2O)

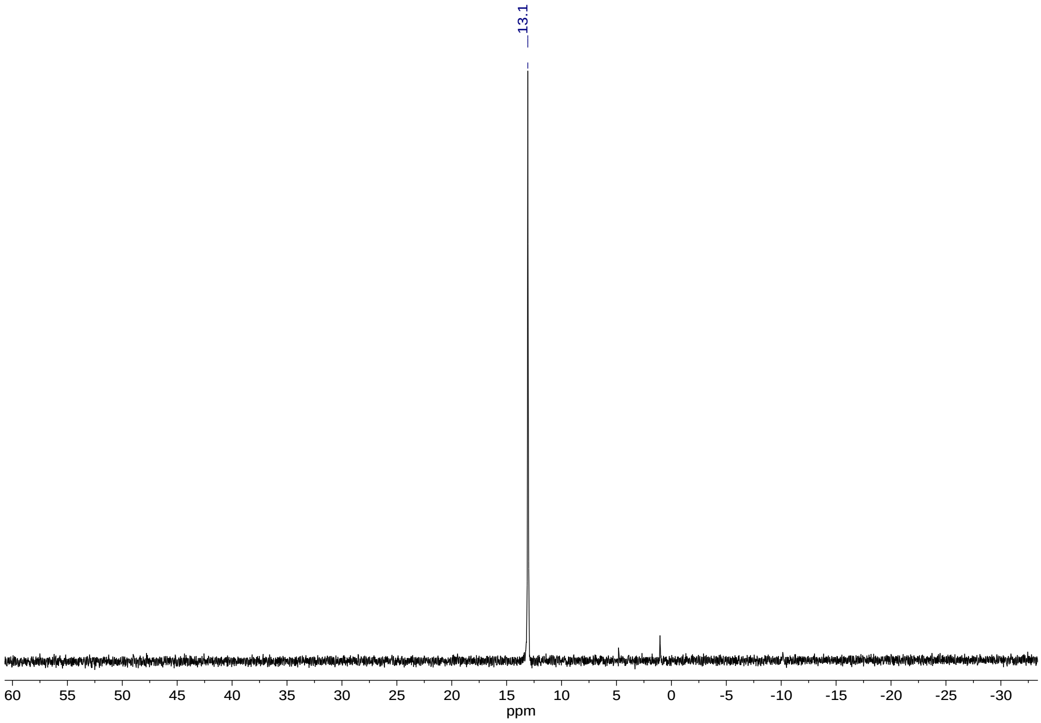

Supplement: Supplementary file 1 [file molecules-26-01654-s001.zip › molecules-1142725-supplementary.pdf]
